# Awareness and intention to register halal certification of micro and small-scale food enterprises

**DOI:** 10.12688/f1000research.75968.1

**Published:** 2022-02-11

**Authors:** Hirawati Oemar, Endang Prasetyaningsih, Siti Zakiah Abu Bakar, Djamaludin Djamaludin, Anis Septiani

**Affiliations:** 1 Industrial Engineering, Universitas Islam Bandung, Bandung, West Java, 40611, Indonesia; 2 Production and Operational Management, Universiti Utara Malaysia, Sintok, Kedah, 06010, Malaysia

**Keywords:** halal awareness, intention, halal certification, food micro and small enterprise, Islam

## Abstract

**Background: **This paper discusses halal awareness of food micro and small-scale enterprises (food MSEs) in West Java Province, Indonesia. Halal awareness is the first step toward obtaining halal certificates, which confirm that the product is lawful according to Islamic Sharia. Unfortunately, most of the food sold on the market do not have halal certificates due to a lack of halal awareness and intention on the part of the entrepreneurs.

**Methods:** This study aims at measuring the level of halal awareness and the intention of food MSE entrepreneurs to register halal certification. Halal awareness is assumed to be influenced by knowledge of halal and MSEs' entrepreneurial perceptions of the benefits of halal certificates. Furthermore, halal awareness, attitudes, and perceptions of ease of procedures will encourage the intention to register halal certification. An electronic Google Form with a cover letter and a set of questionnaires was distributed to collect data. Structural Equation Modelling – Partial Least Square (SEM-PLS) was chosen to evaluate the adopted theoretical models in the exploratory research.

**Results:** The results show that halal awareness is influenced by knowledge of halal and perceptions of benefits. Moreover, halal awareness influences positively the intention to obtain a halal certificate, but the intention is not significantly affected by attitudes and perceptions of procedures for obtaining halal certification. This shows that halal awareness will increase the intention to register halal certification. However, it does not impact attitudes/actions to register for halal certification due to the misconceptions about the procedures for obtaining halal certificates.

**Conclusions:** Micro and small entrepreneurs in West Java Province, Indonesia have a good level of awareness about halal food. However, their products are not halal-certified due to the perceptions of the procedures for obtaining halal certificates, which are relatively complicated and costly for micro and small-scale businesses.

## Introduction

Recently, the halal industry is a fast-growing market globally with a growth rate of 20% per year due to the average growth of the global Muslim population of 1.8%. ^1^ There are approximately 1.6 billion Muslims in the world's population, who live in over 100 countries, and this figure is expected to rise to 2.2 billion by 2030. ^2^

The globalization of the halal industry provides an opportunity for local micro and small-scale enterprises (MSEs) to sell halal products. Local MSEs, on the other hand, are hesitant to compete in the global market because they lack halal certificates, which are one of the most important requirements for entering the global market. Halal certifications are also regarded as a quality-control standard among Muslim consumers. Many non-Muslims have no qualms about eating halal food, but they may react negatively if they eat halal food accidentally and feel cheated. ^3^ As a result, MSEs that want to enter the global market must have a halal certificate.

Halal certificate or halal logo is an important consideration when consumers of both Muslim and non-Muslim, ^4^^–^^6^ or Muslim gen Z, ^7^ purchase products, especially for products made by non-Muslim producers. ^8^ The halal logo has even been recognized in Japan ^9^ where Muslims are a minority. With the issuance of “Undang-Undang Republik Indonesia No 33 Tahun 2014” (Law of Republic Indonesia No 33/2014 - English) ^10^ regarding halal product guarantees, the halal certification has become a requirement for producers in Indonesia. Halal certification is a symbol of ethical behaviour in the food industry that can help entrepreneurs expand their businesses ^11^ or as a business expansion marketing tool. ^7^

Although Islam is the majority religion in Indonesia, most micro, small, and medium enterprises (MSMEs) do not register their products for halal certification. Only 10% of MSMEs have halal certificates, according to the Association of Food and Beverage Entrepreneurs (GAPMMI) in June 2019. ^12^

In another case, most of the food MSEs in the nearby area of Universitas Islam Bandung (Unisba), which is located in Bandung, the capital of West Java Province, Indonesia, do not have halal certificates, although they serve thousands of Unisba students and employees on a daily basis. They have limited knowledge of halal products and the procedures for obtaining halal certificates, as well as a lack of desire to obtain halal certificates. They are unconcerned about the halal status of the materials or the food they sell. ^13^ These indicate the food MSEs' lack of understanding and awareness of the importance of halal products. Hence, it's critical to assess halal awareness and the intention to obtain halal certificates.

The Indonesian Ulama Council (LPPOM-MUI) is a government-appointed institution that issues halal certificates. Each product will receive a halal certificate or halal logo, after going through a certification process that starts with the procurement of raw materials, processing into finished products, storing, and delivering finished products. According to our observations, the majority of food MSEs entrepreneurs in West Java Province, Indonesia, are from low- to middle-income families who are not well-educated. The problem is that food MSEs entrepreneurs may be unaware that raw materials are being processed, or that the processing method does not meet halal standards, resulting in a low intention to obtain a halal certificate. Hence, this study aims at measuring the level of halal awareness and the intention of food MSE entrepreneurs in West Java Province, Indonesia to register halal certification.

This paper is organized as follows; the literature review, the proposed conceptualizing model, the research method, the results, discussion of the results, the conclusions, and data availability.

## Literature review

Studies of halal awareness have been carried out in the last decade. Research of Giyanti and Indriastiningsih ^14^ combines awareness and intention as a single variable, claiming that awareness/intention is influenced by knowledge of halal, perceived benefits, and perceived procedures. However, Dinev and Hu, ^15^ Bachok *et al.,* ^16^ and Rezai and Teng ^17^ show that customer awareness is a strong predictor of their intention to buy or select a product. The influenced factors of halal awareness in this study are derived from Giyanti and Indriastiningsih, ^14^ Dinev and Hu, ^15^ Bachok *et al.,* ^16^ and Rezai and Teng ^17^ and described in the following explanations.

### Halal

In general terms, halal means permitted, authorized, allowed, lawful, legal, or slick according to Islamic Sharia. ^18^ As described in the Law of Republic Indonesia No. 33/2014 ^10^ Halal products are those that conform to Islamic Sharia (principles). Carrions, blood, pigs, and/or halal animals (e.g., chicken, cow, goat, etc.) slaughtered in a manner inconsistent with Islamic Sharia are all considered non-halal materials. Furthermore, non-halal materials also include intoxicating plants or drinks, material that is harmful to one's health, and microbes contaminated with non-halal materials. Halal encompasses substances ( *dzatihi*), the nature of the substances, processes, processing areas, processing instruments, product storage, product distribution, and serving. ^19^ Based on these explanations, this study defines halal as what is permissible for Muslims to eat, drink, and use under Islamic law.

### Halal awareness

Awareness is defined as the state of being aware: knowledge and understanding that something is happening. ^20^ According to the definition of halal used in this study and the definition of the word awareness in the dictionary, halal awareness is then conceptualized as a process of being aware of what is allowed for Muslims to eat, drink, and use.

The level of halal awareness is influenced by religious beliefs, exposures, the role of halal certification through the halal logo/label, and health-related reasons. ^21^ The gender and marital status of SMEs in Thailand have varying levels of awareness to register halal certification. Halal food certificates are required to increase self-confidence, customer trust, and customer satisfaction, although SMEs in Hat Yai, Thailand are dissatisfied with the poor dissemination of halal hub information. ^22^ Halal transportation and halal warehouses also demonstrate SMEs' level of halal awareness in order to maintain the integrity of halal products stored and shipped. ^23^

Halal awareness does not belong solely to halal product manufacturers. Non-halal restaurant owners in Manila, Philippines are generally aware of the 12 halal certification standards (raw materials, tools and equipment, facilities, buildings, exterior areas, location, halal documentation, staff characteristics, staff policies, pest controls, management responsibilities, and waste management), and the majority are ‘Willing’ to be halal certified. ^24^

According to our observations, the majority of micro and small-scale food entrepreneurs in West Java purchase raw materials and process them into finished products without storing and shipping. As a result, the scope of halal awareness in this study is limited to the awareness of using halal materials and processing in a halal manner.

### Knowledge of halal

In the Merriam-Webster dictionary, knowledge is a fact of knowing something with familiarity gained through experience. ^25^ According to the definition of halal used in this study and the definition of the word knowledge in the dictionary, knowledge of halal is then conceptualized as the process of gaining an understanding of what is permissible for Muslims to eat, drink, and use.

The understanding of halal considers knowledge of the laws relating to halal products described in the Quran and Hadith. In general, all foods are permitted except for those derived from prohibited animals such as pigs, dogs, and carrion, as well as foods and beverages containing alcohol and other toxic or dangerous substances. Slaughter must be carried out in accordance with Sharia, with the intention of doing so in the name of God. ^2^ Allah says in the Quran Surah (chapter) 2 (Al Baqarah) ayah (verse) 173 as follows ^26^:

*He has only forbidden to you dead animals, blood, the flesh of swine, and that which has been dedicated to other than Allah. But whoever is compelled (by necessity), without (willful) disobedience nor transgressing (the limits) then there is no sin on him. Indeed, Allah is Oft-Forgiving, and Most Merciful.*

These halal foods are revealed in Law of Republic Indonesia No. 33/2014 concerning halal product guarantees Based on the previously defined scope of halal awareness, knowledge of halal in this study only includes knowledge about halal/non-halal ( *haram*) materials according to the Quran and Hadith, as well as knowledge about the separation of equipment used to process halal/non-halal ( *haram*) materials.

### Benefit

Merriam-Webster defines benefit as something that produces good effects or that promotes well-being: advantage. ^27^ In this study, benefits are conceptualized as the food entrepreneur's perception of the effect to be gained by producing halal food or having halal certificates and labels. The benefits of producing halal food include increasing market share and competitiveness, ^28^ business growth, ^11^ or business development. ^7^

### Procedures

The procedure is defined as a set of steps that must be completed in a specific order. ^29^ In this study, the procedure is conceptualized as the food entrepreneur's perception of the steps that must be taken to obtain a halal certificate and label. Standards for gaining halal certification in Indonesia are explained in Law of Republic Indonesia No. 33/2014 concerning the guarantees of the halal products, ^10^ whereas in the Philippines it is broken down into 12 standards. ^24^ In this study, food entrepreneurs' perceptions of the procedure for obtaining halal certificates in Indonesia involve the availability of information about the certification process, as well as the fact that the certification process is simple, inexpensive, and quick.

### Attitude and intention to register halal certification

In the Merriam-Webster dictionary, attitude is defined as a way of thinking that influences one's behaviour, ^30^ while the intention is the thing that you plan to do or achieve **:** an aim or purpose. ^31^ In this study, attitude is conceptualized as thinking about producing a halal product, while the intention is conceptualized as an act of registering the product to obtain a halal certificate.

According to the Planned Behavior Theory, the intention is determined by three independent factors, i.e., attitude toward behaviour, subjective norm, and perceived behavioural control. Supposing that there is a positive attitude that is supported by people around (as a subjective norm) and there is a perception of ease to perform the behaviour under consideration (as a behavioural control), then an individual's intention to behave will be higher. ^32^

From an Islamic perspective, every Muslim must have an attitude to like and want to do a good job. In a broader context, attitude means to do good deeds due to Allah (God) loves those who do good as Allah (God) commands in Quran surah (chapter) Al-Baqarah ayah (verse) 195 ^26^:

*And spend in the way of Allah and let not your own hands throw yourselves into destruction. And do good; indeed, Allah loves the good-doers.*

Attitude is also associated with two conditions i.e. good (‘ *mahmudah*’) and bad (‘ *mazmumah*’). ^33^

Several previous studies show that there is a positive relationship between attitude and intention to buy or choose a product. Consumers' attitudes and perceived prices are the most significant factors influencing consumers' purchase intentions for private labeled food products. ^34^ Attitude is also one of the factors that influence the intention to buy branded content on Webisodes, a branded content strategy that provides hidden communication. ^35^ A positive relationship between attitude and purchase intention toward organic foods has been shown by Yang, *et al*. ^36^

This study restricts food entrepreneurs' attitudes including a focus on halal issues, a guarantee of selling halal products, halal product attention, and checking to use halal raw materials. Meanwhile, food entrepreneurs' intentions are limited to being responsive to the certification process, attempting to meet halal quality standards, implementing a halal assurance system, and registering halal certification as soon as possible.

## Conceptualizing halal awareness

### Identification of variables

Motivation to obtain halal-certified is significantly influenced by religious understanding and motivation to gain profit. ^19^ The awareness/intention to get halal certificates is significantly influenced by a motivation to get profit because SME entrepreneurs generally agreed that Halal Food Certification provided benefits. ^14^ Meanwhile, the procedure for obtaining halal certificates is relatively complex, thereby reducing the intention of SMEs to register halal certification. Meanwhile, Lee and Shin ^37^ found that consumers' awareness of Corporate Social Responsibility (CSR) activities and purchase intentions are positively related. According to the Planned Behavior Theory, the intention is influenced by attitude, while the ease of procedure to obtain halal certificates will affect the intention to register their products to get halal certificates.

Referring to Giyanti and Indriastiningsih, ^14^ Waluyo, ^19^ and Lee and Shin, ^37^ and the Planned Behavior Theory, ^32^ this study identifies that the variables are knowledge of halal (KH), perception of benefits (PB), perception of procedures (PP), halal awareness (HA), attitude to produce halal product (AHC) and intention to register a halal certificate. Measurement items of each variable are then compiled from the previous studies. ^10^^,^^14^^,^^19^^,^^21^^–^^23^^,^^28^

### Conceptual model and hypothesis

**Figure 1.**
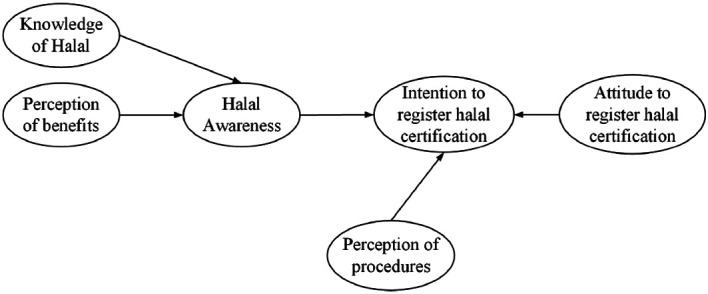
Conceptual model of awareness and intention to register halal certification.

Referring to Waluyo ^19^ and Giyanti and Indriastiningsih, ^14^ this study considers that halal awareness is influenced by knowledge of halal and MSEs perception of benefit on the halal certificate. Referring to the Planned Behavior Theory, the halal awareness and attitude (as a positive attitude) and MSEs' perceptions of ease of procedures (as a behavioural control) will encourage the intention for registering halal certification. The relationship of these variables represents the conceptual model of halal awareness and intention to halal certification (see Figure 1).

As can be seen in Figure 1, halal awareness and intention to register halal certificates involve variables of knowledge of halal, perceptions of benefits, perceptions of procedures, and attitudes. Halal awareness is an independent variable, while knowledge of halal and perceptions of benefits are dependent variables. Furthermore, halal awareness, perception of procedures, and attitude to register halal certification are dependent variables, while the intention to register halal certification is an independent variable.

As can be seen in the conceptual model ( Figure 1), the level of halal awareness is influenced by the knowledge of halal which may include MSEs' understanding of the types of non-halal food as mentioned in Law of Republic Indonesia No. 33/2014. ^10^ Therefore, we hypothesize that:

*H1: The knowledge of halal/non-halal levels (KH) positively affects Halal Awareness (HA) of food MSEs.*

Halal certificates and halal logos are perceived to have benefits in increasing consumer confidence, ^22^ to use as a promotional tool, ^14^ and compete with other producers. ^22^ Hence, halal certificates are expected to improve the MSEs performance. Based on this point of view, we hypothesize that:

*H2: Perception of benefits (PB) positively affects Halal Awareness (HA).*

Halal awareness is measured by awareness of the importance of using halal materials in producing halal products, ^24^ and perceiving the benefits to be gained despite the process is very strict. ^22^

Consumer awareness of the green concept is a strong predictor of their intention to consume green foods, ^17^ while customer awareness towards the halal logo fosters purchase intention. ^16^ Referring to Ambali and Akbar ^38^ and Ngah, *et al*. ^23^ the intention considers regulating actions and a sense of responsibility to gain a halal certificate. In this study, halal awareness is expected to influence on intention to register halal certification. Hence, we hypothesize that:

*H3: Halal Awareness (HA) positively affects Intention to register Halal Certification (IHC).*

MSE entrepreneurs perceive that the procedure to achieve halal certification is complex due to the lack of information from respondents regarding halal certification procedures. ^14^ This will negatively influence the intention of producers to register halal certification. In the light of this, we hypothesize that:

*H4: The MSEs' perceptions of ease of the procedures (PP) positively affect the Intention to register halal certification (IHC).*

A positive relationship between attitude and intention has been shown by Jaafar, *et al.,* ^34^ Yang, *et al.,* ^36^ and Rezai, *et al.* ^17^ These show that attitude influences intention. Hence, we hypothesize that:

*H5: Attitude to Halal Certification (AHC) positively affects the Intention to register halal certification (IHC).*

## Methods

### Study design and participants

This study adopts a quantitative method to evaluate the hypothesis, i.e., analyze the data using descriptive statistics. This study has followed the STROBE guidelines/checklist for cross-sectional research.

Initially, 680 micro and small-scale food and beverage entrepreneurs in the West Java MSME community were targeted. However, based on our observations, medium-scale enterprises differ from micro and small enterprises in terms of the entrepreneurs' education and economic status. Medium-scale entrepreneurs have a better education and economy than MSEs. This has an impact on knowledge and understanding of the significance of halal-certified products. Because micro and small-scale entrepreneurs are more profit-driven, regardless of halal or haram, many products on the market are uncertified. Hence, this research focuses on MSEs. Furthermore, we separate the food and beverage enterprises, due to the different halal requirements for raw materials. Finally, the unit of analysis for this research is food MSEs who are members of the MSME group rather than food and beverage MSME.

The respondent criteria are 1) micro and small-scale food entrepreneurs with an annual sales turnover are less than 2,500 Rupiahs and net assets are less than 500 million Rupiahs (see Table 1); 2) have an ongoing business; 3) do not have a halal certificate. A Google Form with a cover letter and a set of questionnaires were sent out to potential respondents. This method was chosen because of the COVID-19 pandemic outbreak. In addition, the designed questionnaires could be collected without conducting direct visits to the respondents. The respondents could not participate in the survey unless they gave their written consent. Data from food entrepreneurs were collected from March to May 2020.

**Table 1.** Category of micro, small and medium business in Indonesia.

Category	Net assets (in million Rp.)	Annual sales turnover (in million Rp.)
Micro	50 (max)	300 (max)
Small	50 – 500	300 – 2,500
Medium	500 – 10,000	2,500 – 50,000

Source: Law of the Republic of Indonesia No. 20/2008. ^40^

We received written permission from the Chairman of the West Java MSME associations to contact these food entrepreneurs in West Java for data collection. To guarantee that there is no conflict of interest in this study, survey responses are kept anonymous.

### Data collection

Data were collected by sending electronically to the respondent standard questionnaires modified from previous studies. The questionnaires were re-translated from English to Indonesian, except for those referring to Waluyo ^19^ due to already being in Indonesian. A copy of the distributed questionnaires can be found in Extended Data. ^39^

The questionnaire was pretested with a small sample of food MSE entrepreneurs before being distributed to the actual respondents. Based on pretest feedback, the wording of some items was refined and modified to guarantee that the validity and reliability of each variable meet the required standard. The question items were scored on a Likert scale of 1 (strongly disagree) to 5 (strongly agree). The follow-up of this plan is described later in the goodness of measurements instruments session.

The measurement items for the knowledge halal variable were adapted from Giyanti and Indriastiningsih ^14^ and Waluyo. ^19^ The survey section includes four items, such as knowledge of; slaughtering methods (KH1), haram products (KH2 and KH3), and processing equipment (KH4).

The measurement items for the perceptions of benefits variable were adapted from Giyanti and Indriastiningsih ^14^ and Abdul. ^22^ These items asked about respondents' perceptions of the benefits they would get if they had a halal certificate, such as a promotional tool (PB1), more convincing consumers to buy (PB2), improving business performance (PB3), and more competitive (PB4).

The measurement items for the perceptions of the procedure variable were adapted from Giyanti and Indriastiningsih ^14^ and Ambali and Bakar. ^21^ This item asked about the respondent's perception of the procedure for obtaining halal certificates in Indonesia, such as the existence of information about the certification process (PP1), the certification process is easy (PP2), cheap (PP3), and fast (PP4).

The measurement items for the halal awareness variable were adapted from Abdul ^22^ and Law of Republic Indonesia No 33/2014. ^10^ This part of the survey asked about the awareness of respondents about the importance of producing halal food (HA1), using halal raw materials (HA2), the importance of having a halal certificate (HA3), and the rigorous of the halal certification process (HA4).

The attitude variable measurement items to produce halal products were adapted from Ambali and Bakar ^21^ and Menteri Hukum dan HAM. ^10^ These items include respondents' attitudes to always pay attention to halal issues (AHC1), ensure consumers buy halal products (AHC2), pay attention to halal products (AHC3), and use halal materials (AHC4).

The measurement items for the variable of intention to register halal certification were adapted from Ngah, *et al*., ^23^ Abdul, *et al*., ^28^ and Law of Republic Indonesia No 33/2014. ^10^ These items include respondents' intention to be responsive to the certification process (IHC1), strive to meet halal quality standards (IHC2), immediately implement halal assurance system (IHC3), and immediately register halal certification (IHC4). The variables, indicators, and measurement items are described in Table 2.

**Table 2.** Variables, indicators, and measurement items of halal awareness.

Variables	Indicators	Measurement Items	References
Knowledge of Halal (KH)	KH1	Halal animals are slaughtered not following Islamic Sharia is non-halal ( *haram*)	^19^
	KH2	Products containing alcohol used in the production process are non-halal ( *haram*)	^14^
	KH3	Pork and its derivation used in the production process are non-halal ( *haram*)	
	KH4	Equipment used to produce halal food must be kept separate from equipment used to produce non-halal food	
Perception of Benefits (PB)	PB1	Halal certificate can be used as a promotional tool	^14^
	PB2	The ownership of Halal certificate increases consumer trust in MSE products.	^22^
	PB3	The Halal certificate contributes to the development of MSE performance	
	PB4	The halal certificate will make MSEs more competitive	
Perception of Procedures (PP)	PP1	The MSE has sufficient information on the halal certification process	^21^
	PP2	Halal Certification is a relatively simple process.	^14^
	PP3	The cost of maintaining halal certification is cheap for MSE	
	PP4	The time of obtaining halal certification is relatively quick	
Halal Awareness (HA)	HA1	The MSE is aware of the importance of producing halal food	^10^
	HA2	The MSE is aware of the importance of using halal raw materials	
	HA3	The MSE is aware of the importance of a halal certificate	^22^
	HA4	The MSE is aware of the rigorous certification process.	
Attitude to produce halal product (AHC)	AHC1	The MSE is always concerned about a product's halal issue	^21^
	AHC2	As a food producer, the MSE is always concerned that its customers purchase products that follow Islamic Sharia.	
	AHC3	The MSE is always concerned with producing halal products.	
	AHC4	The MSE ensures that the raw materials are halal at all times.	^10^
Intention to Register Halal Certification (IHC)	IHC1	Although the MSE ensured that halal materials were used, the MSE is still in charge of halal certification	^28^^,^^23^
	IHC2	The MSE must try to comply halal quality standards to obtain halal certification	
	IHC4	The MSE will register the products of MSEs to obtain halal certification	
	IHC3	The MSE will apply the Halal assurance system in their business	^10^

We analyzed the chi-square of early and late respondents' responses to see if there was any potential for non-response bias (the first and last 20 percent of responses received). The findings show that there is no significant variation in responses on key metrics between early and late respondents.

### Structural model analysis

Data are analyzed with descriptive statistics to provide a description of the respondents' profile, the results of the assessment of the level of knowledge of halal, perceptions of benefits, halal awareness, perceptions of procedure, attitude, and intention to register halal certificates. The research analysis is intended to assess the model and to objectively describe the hypotheses.

The adopted theoretical models are evaluated using Structural Equation Model-Partial Least Square (SEM-PLS) method because this study is exploratory research to predict certain constructs by focusing on explaining the variance in the dependent variables when examining the model. ^41^ Free Smart-PLS software 3.3.2. is chosen as data processing.

### The goodness of the measurement instrument

In this study, both validity and reliability tests are carried out to measure the goodness of the shared questionnaires. Validity is a test of how well the developed instrument measures the particular construct being measured, while reliability is a test of how the developed instrument consistently measures the construct being measured. ^42^

Initially, the questionnaires had been planned to be pretested in a small sample of food MSEs by distributing it directly beginning in March 2020. However, we were unable to meet with the entrepreneurs due to the social distancing caused by the COVID-19 outbreak. Finally, we decided to distribute the electronic questionnaire Google Forms in May 2020. As a result, the data collected reached 100 respondents in just a matter of days. Due to the limitations of the software features used, the distribution of the questionnaire was halted. As a result, all collected data was subjected to validity and reliability tests.

### Measure for validity

In this study, measuring validity uses construct, convergent and discriminant validity to check how well the questionnaire measures the constructs that fit with the adopted theory.

1. Construct validity

The construct validity is assessed by looking at loading and cross-loading to identify problem items if there are any. The validity test using cross-loading is patterned that the main loading factor originating from its construct is greater than the correlation value built from these variables on other constructs. ^43^ Table 3 presents an evaluation of validity based on the value of the main loading factor to the value of cross-loading factors with other constructs. As shown in Table 3, the value of the main loading factor of each construct is higher than the value of the loading factor outside of the main loading factor, so it can be concluded that all constructs are declared valid. For example, the loading factor of AHC1 is higher than the loading factor of HA, IHC, KH, PB, and PP.

**Table 3.** Cross loading for construct validity.

	AHC	HA	IHC	KH	PB	PP
AHC1	0.911	0.781	0.754	0.703	0.674	0.313
AHC2	0.917	0.824	0.760	0.733	0.770	0.187
AHC3	0.946	0.922	0.825	0.759	0.834	0.173
AHC4	0.853	0.722	0.701	0.581	0.620	0.268
HA1	0.857	0.942	0.800	0.751	0.811	0.097
HA2	0.876	0.955	0.817	0.767	0.831	0.153
HA3	0.805	0.941	0.880	0.693	0.871	0.190
HA4	0.722	0.787	0.718	0.541	0.686	0.218
IHC1	0.732	0.757	0.909	0.553	0.758	0.239
IHC2	0.857	0.885	0.952	0.680	0.848	0.197
IHC3	0.775	0.848	0.948	0.706	0.833	0.187
IHC4	0.729	0.778	0.885	0.601	0.712	0.185
KH1	0.641	0.634	0.590	0.927	0.576	0.151
KH2	0.578	0.540	0.531	0.797	0.458	0.188
KH3	0.672	0.710	0.621	0.924	0.640	0.085
KH4	0.722	0.698	0.610	0.773	0.650	0.205
PB1	0.781	0.832	0.804	0.697	0.909	0.201
PB2	0.801	0.855	0.842	0.653	0.942	0.190
PB3	0.718	0.829	0.774	0.594	0.924	0.187
PB4	0.648	0.736	0.725	0.582	0.914	0.243
PP1	0.234	0.183	0.199	0.202	0.215	0.829
PP2	0.265	0.179	0.233	0.122	0.220	0.933
PP3	0.227	0.150	0.188	0.161	0.189	0.902
PP4	0.161	0.102	0.129	0.174	0.145	0.931

2. Convergent validity

Convergence validity is evaluated by comparing loading factor with both Composite Reliability (CR) and Average Variance Extracted (AVE). ^41^^,^^43^ The acceptance level for loading factors is more than 0.5, while the acceptance level for CR is more than 0.70, and the AVE value of 0.50 or higher. Table 4 shows that all items’ loadings exceed 0,5, while all CR values are higher than 0.7. In this study, the AVEs for the indicators are within the range of 0.736 and 0.854 respectively ( Table 4). Hence, the questionnaire fulfills the requirements for convergent validity. Based on Table 4 it can be concluded that all constructs have *p*-values smaller than 5% as suggested by Budhiasa, ^43^ so it can be stated that all constructs are valid.

**Table 4.** Result of the measurement model.

Variables	Indicator	Loading factor	*p*-value	CR	AVE
KH	KH1	0.927	0	0.917	0.736
	KH2	0.797	0		
	KH3	0.924	0		
	KH4	0.773	0		
PB	PB1	0.909	0	0.958	0.851
	PB2	0.942	0		
	PB3	0.924	0		
	PB4	0.914	0		
HA	HA1	0.942	0	0.95	0.826
	HA2	0.955	0		
	HA3	0.941	0		
	HA4	0.787	0		
PP	PP1	0.829	0	0.942	0.802
	PP2	0.933	0		
	PP3	0.902	0		
	PP4	0.913	0		
IHC	IHC1	0.909	0	0.959	0.854
	IHC2	0.952	0		
	IHC3	0.948	0		
	IHC4	0.885	0		
AHC	AHC1	0.911	0	0.949	0.823
	AHC2	0.917	0		
	AHC3	0.946	0		
	AHC4	0.853	0

3. Discriminant validity

The square root of the AVE of each construct should be greater than its highest correlation with any other construct, according to the Fornell-Larcker criterion. ^41^ Furthermore, the outer loadings of an indicator on a construct should be greater than all of its cross-loadings with other constructs. Table 5 shows that the average variance extracted by the indicators measuring that construct is less than the squared correlations for that construct. To put it another way, the measurement model represents adequate convergent and discriminant validity.

**Table 5.** Discriminant validity construct.

	AHC	HA	IHC	KH	PB	PP
AHC	**0.907**					
HA	0.899	**0.909**				
IHC	0.839	0.887	**0.924**			
KH	0.763	0.761	0.69	**0.858**		
PB	0.802	0.884	0.855	0.687	**0.923**	
PP	0.256	0.179	0.218	0.181	0.221	**0.895**

### Measure for Reliability

The Reliability test is evaluated by the Alpha Cronbach value, the most widely used test. The Alpha Cronbach value higher than 0.6 is acceptable in exploratory research. ^41^ As shown in Table 6, the Alpha Cronbach’s are within the range of 0.878 and 0.943, hence it confirms the reliability of the instrument.

**Table 6.** Reliability test result.

Variable	Alpha Cronbach
KH	0.878
PB	0.942
HA	0.928
PP	0.918
IHC	0.943
AHC	0.928

### Ethical consideration

The Ethical Licensing Committee of the Islamic University of Bandung approved this study by Protocol number: 495/B.04/Bak-k/XII/2019. We had provided all respondents with a consent statement after consultation. The written consent to participate from the Chairman of the West Java MSME Community was gained according to document number: 015/SKIP/IV/2020. In the questionnaire, there is a statement that by filling out the questionnaire the respondents gave their consent to participate. Respondents gave their consent to take part when they filled out the questionnaire. Respondents had given their consent truly and without coercion. Furthermore, to protect respondents' rights and privacy, all forms of data obtained will be kept confidential.

## Results

### Structural analysis

Barclay, Higgins, and Thompson (1995) in Hair *et al*. ^41^ explain the Ten Times Rule in determining the number of PLS-SEM samples, which states that the sample size must be greater than (1) ten times the greatest number of formative indicators used to measure a single construct, or (2) ten times the greatest number of structural paths directed at a specific construct in the structural model. In other words, the minimum sample size is equal to 10 times the maximum number of arrows in the PLS path model pointing to the latent variable. ^41^

In this study, the IHC variable is the latent variable with the maximum number of arrows, i.e., 3 (see Figure 1). As a result of the Ten Times Rule, 3.10 = 30 represents the bare minimum of observations required to estimate the PLS path model depicted in Figure 1. In terms of Cohen's (1992) recommendation for multiple OLS regression analysis, or running a power analysis using the G*Power program, as cited in Hair, *et al.,* ^41^ 33 observations are required to detect an *R ^2^* value of about 0.25, assuming a statistical power of 80% and significance level of 5%.

### Participants

The questionnaires were distributed electronically to the West Java MSME community. The questionnaires were returned by 376 people. The returned questionnaires were then sorted using predetermined criteria, yielding 137 questionnaires that met the criteria. However, due to the limitations of the software features used, this study only processed 100 questionnaires at random.

Table 7 displays the percentage of respondents for each indicator. As shown in Table 7, 98% of respondents have Islam as their religion (Muslim), 71% are female, and 88% are 26 years old or older. In terms of business size, 89% of respondents are micro-scale entrepreneurs.

**Table 7.** Respondent’s profile.

Indicator		Quantity	Percentage
Religion	Islam	98	98%
	Catholic	1	1%
	Protestant	1	1%
Gender	Male	29	29%
	Female	71	71%
Age	<17 years	1	1%
	17-20 years	6	6%
	21-25 years	7	7%
	26-40 years	33	33%
	>40 years	53	53%
Business scale	Micro	89	89%
	Small	11	11%

### Descriptive statistics of the research variables

Table 8 shows the descriptive statistics for each measurement indicator. The knowledge of halal (KH) has a high average perception value.

**Table 8.** Descriptive statistics measurement indicators.

Indicator	Minimum	Maximum	Mean	Total mean	Deviation standard
KH1	1	5	4.71	**4.675**	0.816
KH2	1	5	4.51		0.995
KH3	1	5	4.79		0.739
KH4	1	5	4.69		0.784
PB1	1	5	4.68	4.668	0.705
PB2	1	5	4.73		0.719
PB3	1	5	4.66		0.79
PB4	1	5	4.6		0.812
HA1	1	5	4.73	4.64	0.676
HA2	1	5	4.77		0.661
HA3	1	5	4.69		0.717
HA3	1	5	4.37		0.913
PP1	1	5	3.4	3.2225	1.158
PP2	1	5	3.24		1.176
PP3	1	5	3.18		1.169
PP4	1	5	3.07		1.243
IHC1	1	5	4.57	4.625	0.725
IHC2	1	5	4.63		0.73
IHC3	1	5	4.66		0.738
IHC4	1	5	4.64		0.755
AHC1	1	5	4.6	4.635	0.787
AHC2	1	5	4.68		0.747
AHC3	1	5	4.73		0.676
AHC4	1	5	4.53		0.846

### Hypothesis testing

**Figure 2.**
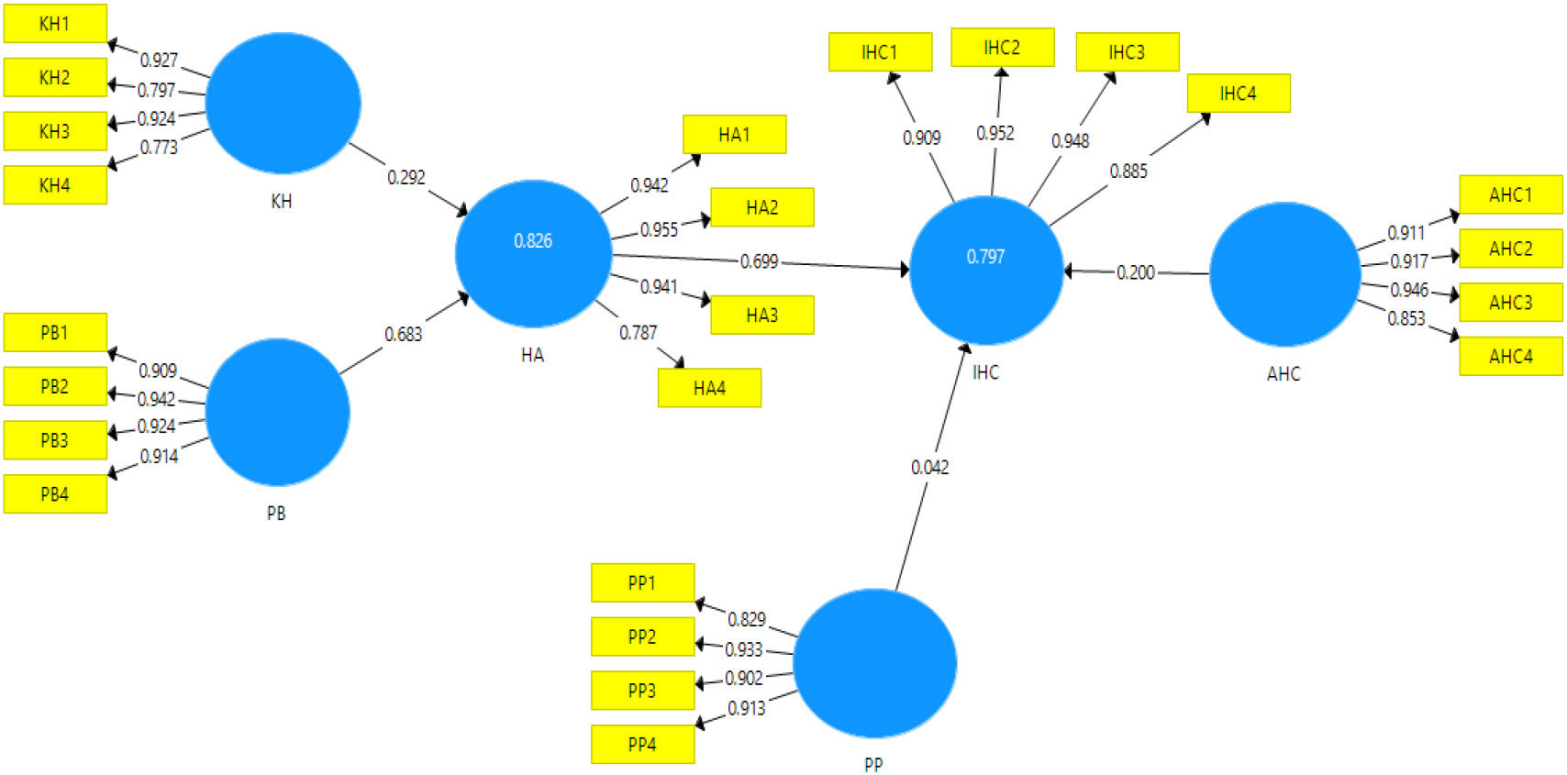
Result of path analysis.

Figure 2 shows the path analysis result of halal awareness and the intention to register halal certification using free Smart PLS 3.3.2. software, while Table 9 represents the hypothesis testing results of each path.

**Table 9.** Path coefficients and hypothesis testing results.

Hypothesis	Relationship	Coefficient	*t*-value	*p*-value	Remark
H1	KH-HA	0.292	3.01	0.003	Significant
H2	PB-HA	0.683	7.646	0	Significant
H3	HA-IHC	0.699	4.071	0	Significant
H4	PP-IHC	0.042	0.989	0.323	Not significant
H5	AHC-IHC	0.200	1.119	0.264	Not significant

In this section, the path analysis is addressed to ascertain the hypotheses put forward. As can be seen in Figure 2, the *R ^2^* value of 0.797 for IHC indicates that 79.7% of the variance in IHC can be explained by HA, PP, AHC. In addition, Figure 2 also shows that HA, PP, and AHC are positively related to IHC among MSEs entrepreneurs with β = 0.699, 0.042, and 0.2, respectively. According to the *t*-value of the path coefficients, HA has a significant impact on IHC, while AHC and PP do not have a significant impact on IHC.

Besides, the *R ^2^* value of 0.826 for HA means that 82,6% of the variance in HA is influenced by KH and PB, with values β = 0,292 and 0,683 respectively. Hence, KH and PB have a strong and significant impact on the awareness of MSE entrepreneurs about the importance of halal products. According to Hair, *et al.* ^41^ *R ^2^* values of 0.75, 0.50, or 0.25 for the endogenous construct, respectively, can be described as substantial, moderate, or weak. The *R ^2^* value of an endogenous latent variable (i.e., halal awareness) described by the two predictive constructs in this study is 82.6% (see Figure 2), which is substantial. Hence, KH and PB are genuine predictors of MSEs awareness to produce a halal product. The same goes for the *R ^2^* value of the endogenous latent variable (i.e., Intention to register halal certification) is 79,7% which is substantial. However, the genuine predictor of MSEs Intention to register halal certification is only HA.

## Discussion

As can be seen in Table 8, the findings of the descriptive statistics indicate that most of the respondents are highly aware to have a halal certificate because they have a high level of knowledge of halal and generally agree that Halal Food Certification provides benefits. These findings have confirmed the findings of Waluyo, ^19^ that the religious knowledge and motivation to benefit have a significant impact on the awareness of food producers to certify their product. The finding that knowledge of halal has a significant impact on knowledge of halal is different from the finding of Giyanti and Indriastiningsih ^14^

As shown in Figure 2, awareness of halal certification and intention to register halal certification have a correlation coefficient of 0.699. It means that the awareness of halal certification is a strong indicator of the intention to register halal certification. It aligns with Rezai, *et al.* ^17^ and Bachok, *et al.* ^16^ The descriptive statistics also show that most of the respondents have a high intention to register halal certification with a total mean of 4.625 on a 5 scale ( Table 8).

This study finds that attitudes to produce halal products and perceptions of halal certification procedures have a positive correlation with intentions, but both do not significantly affect intentions. According to the Planned Behavior Theory, when there is support and a sense of ease that there are no barriers to behaviour, intention to behave will increase. ^44^ This study shows that intention to register a halal certificate is not supported by the attitude because West Java food MSEs perceive the procedures to obtain halal certification to be complex.

The findings also show that MSEs are aware of the benefits of the halal certificate. Based on our observation, we find that they do not require a halal certificate because they have satisfied with the sales/performance that has been achieved.

Another factor that hinders the desire to obtain a halal certificate is the lack of consumer pressure. Furthermore, MSEs are unaware of the risks of violating the halal product guarantee law if they do not have a halal certificate. Food MSEs' entrepreneurs' lack of understanding of the benefits and risks of having a halal certificate is alleged to lead to that attitude having little bearing on their desire to register for halal certification

These are the study's main findings. We observe that the local community culture and mindset of micro and small-scale food entrepreneurs appear to be driving this lack of intention to obtain a halal certificate. In general, West Java food MSEs are low- to middle-income communities with limited educational opportunities, so they rarely have broad perspectives or are willing to progress and develop. It is already a good thing that they can sell every day without having to develop their business. They are unaware that halal certificates will increase consumer trust and help food businesses compete more effectively. Halal certificates will therefore increase sales and revenue. Furthermore, by selling halal products, they have aided people who follow the Islamic faith to practice the Quran surah (chapter) 2:(Al Baqarah, ayah (verse) 168 ^26^:

*O mankind! Eat from whatever is on the earth - lawful and good and do not follow the footsteps of Shaitaan devil. Indeed, he is your clear enemy.*

MSEs' perceptions of complicated and costly certification procedures for their scale of operation also hampered their desire to register for halal certification. This finding supports Giyanti and Indriastiningsih, ^14^ whose study found that MSEs' food awareness/intention is hampered by a lack of socialization and the complexity of the procedure for handling halal certification. As a result, socialization and training on halal certification procedures will raise the level of intention.

Socialization and training on halal awareness, halal guarantee system, and halal certification for street vendors around Universitas Islam Bandung (Unisba) which is located at Bandung, the Capital of West Java Province Indonesia have been conducted by Oemar *et al.* ^13^ The trainees gain a better understanding and awareness of halal food as a result of the training. Consequently, all trainees intend to obtain a Halal Certificate. However, there are several barriers to obtaining halal certificates, including costs (68%), time (24%), and procedures (28%).

This research could aid efforts to increase food entrepreneurs' desire to obtain halal certifications. Micro and small businesses must be educated about the benefits of obtaining halal certificates and the ease with which they can do so. Micro and small food entrepreneurs' halal awareness should be reinforced by the availability of a readily accessible halal information centre and innovative ecosystems.

## Conclusions

It can be concluded that micro and small entrepreneurs in West Java Province, Indonesia have a good level of awareness about halal food even though they do not have a halal certificate. They pay attention to the halalness of the material used and of its processing. However, the perceptions of the procedures to obtain halal certificates which are relatively complicated and expensive for micro and small-scale businesses discourage the MSEs to register halal certification.

The hypothesis test shows that knowledge and perceptions of benefit have positive and significant correlations to halal awareness. In addition, halal awareness, attitude, and perception of the procedure have a positive influence on the intention to register halal certification. However, attitude and perception of procedure do not have a significant impact, while halal awareness has a significant effect on intention. This shows that halal awareness of micro and small-scale food entrepreneurs can increase intention to register their products to be halal certified. However, the reality shows that many products sold in the market do not have halal certificates. It indicates that the halal awareness of micro and small-scale entrepreneurs does not have an impact on real actions to register halal certification. They will take action when they gain real benefit/profit.

Finally, we recognize that this study has some limitations, including 1) The study focuses on micro and small food businesses. Perhaps in the future, research can be done for medium-scale food businesses that have a unique character due to the entrepreneur's higher level of education. 2) The research was conducted in West Java, which has a distinct culture and mindset. In the future, perhaps research can be conducted in other Indonesian provinces with different characteristics. 3) The research was conducted for the food industry. Perhaps, study on other products will be possible in the future. 4) There are six variables in this study. Other estimated variables may be added in the future.

Further research may be undertaken to measure the level of halal awareness and intention to obtain the halal certificate for medium-scale entrepreneurs with other kinds of products in the other provinces and consider other related variables.

## Data availability

### Underlying data

Figshare: Dataset of Questionnaire Result from the respondents of Awareness to Register Halal Certification https://doi.org/10.6084/m9.figshare.17078381.v1. ^45^

This project includes the following underlying data:

- Questionnaire results from 100 West Java micro and small food entrepreneurs.

Data are available under the terms of the Creative Commons Attribution 4.0 International license (CC-BY 4.0).

### Extended data

Figshare: List of questions of descriptions of the questionnaire - Awareness to Register Halal Certification. https://doi.org/10.6084/m9.figshare.17078336.v1. ^39^

This project includes the following extended data:

- A copy of the questionnaire

Data are available under the terms of the Creative Commons Attribution 4.0 International license (CC-BY 4.0).

Figshare: The Respondent characteristics of Awareness to Register Halal Certification. https://doi.org/10.6084/m9.figshare.17078354.v1. ^46^

This project includes the following extended data:

- Profile of respondents

Data are available under the terms of the Creative Commons Attribution 4.0 International license (CC-BY 4.0).
