# Awareness and intention to register halal certification of micro and small-scale food enterprises

**DOI:** 10.12688/f1000research.75968.3

**Published:** 2023-03-29

**Authors:** Hirawati Oemar, Endang Prasetyaningsih, Siti Zakiah Abu Bakar, Djamaludin Djamaludin, Anis Septiani

**Affiliations:** 1 Industrial Engineering, Universitas Islam Bandung, Bandung, West Java, 40611, Indonesia; 2 Production and Operational Management, Universiti Utara Malaysia, Sintok, Kedah, 06010, Malaysia

**Keywords:** halal awareness, intention, halal certification, food micro and small enterprise, Islam

## Abstract

**Background: **This paper discusses halal awareness of MSE food producers in West Java Province, Indonesia. Halal awareness is the first step toward obtaining halal certificates, which confirm that the product is lawful according to Islamic Sharia. Unfortunately, despite Islam being the religion of most Indonesians,

most food sold in the market lacks a halal certificate due to a lack of awareness among food producers about the importance of selling halal-certified foods.

**Methods: **This study aims at measuring the level of halal awareness and the intention of MSE food producers to register halal certification. Halal awareness is assumed to be influenced by knowledge of halal and the food producers’ perception of the benefits of halal certificates. Furthermore, halal awareness, attitudes, and perception of the ease of procedures will encourage the intention to register halal certification. An electronic Google Form with a cover letter and a set of questionnaires was distributed to collect data. Partial Least Square - Structural Equation Modelling (PLS-SEM) was chosen to evaluate the adopted theoretical models in the exploratory research.

**Results:** The results show that halal awareness is influenced by knowledge of halal and perception of its benefits. Moreover, halal awareness influences positively the intention to obtain a halal certificate, but the intention is not significantly affected by attitudes to produce halal foods and perception of procedures for obtaining halal certification. This shows that halal awareness will increase the intention to register halal certification. However, misconceptions about the procedures for obtaining halal certificates keep them from registering.

**Conclusions:** MSE food producers in West Java Province, Indonesia have a good level of awareness about halal food. However, their products are not halal-certified due to the perception of the procedures for obtaining halal certificates are relatively complicated and costly for them.

## Introduction

A halal certificate is a symbol of ethical behaviour in the food industry that can help entrepreneurs expand their businesses. ^1^ ^,^ ^2^ Halal certificate or halal logo is regarded as a quality-control standard which is an important consideration when consumers of both Muslim and non-Muslim, ^3^ ^–^ ^5^ or Muslim gen Z, ^2^ purchase products, especially for products made by non-Muslim producers. ^6^ Many non-Muslims have no qualms about eating halal food, but they may react negatively if they eat accidentally halal food and feel cheated. ^7^ The halal logo is even recognized in Japan, ^8^ where Muslims constitute a minority. Furthermore, non-halal restaurant owners in Manila, Philippines are generally aware of the halal certification standards and are ‘Willing’ to become halal certified. ^9^ These studies show the necessity of halal certificates for a product.

Indonesia is a country where Islam is the majority religion. The Indonesian government has mandated that food producers have halal certificates to protect consumers, particularly Muslim consumers when purchasing food, by issuing Undang-Undang Republik Indonesia No 33 Tahun 2014" (Law of Republic Indonesia No 33/2014 - English) ^10^ regarding halal product guarantees. The mandatory halal for food and beverages will be enforced starting October 17, 2024. However, statistics show that the majority of food micro, small, and medium-scale enterprises (food MSMEs) do not register their products for halal certification. According to the Association of Food and Beverage Entrepreneurs (GAPMMI) in June 2019, only 10% of MSMEs have halal certificates. ^11^ This contradiction indicates that the intention of SMEs to sell halal-certified products remains low. They may be unaware of the benefits of halal certification or have a negative perception of halal certificates. Several studies on the perception of halal certificates have been conducted in various cities in Indonesia.

The majority of MSME entrepreneurs in some cities of East Java Province, Indonesia, such as Bangkalan, Pamekasan, and Pasuruan, understand the significance of halal certificates. However, they consider halal certification to be unimportant due to the lack of socialization, complicated requirements, high cost of the halal certification process as well as lack of assistance. ^12^ In East Kalimantan Province, Indonesia the MSME entrepreneurs even underestimate the halal certificate due to believing that their business is running smoothly despite the lack of a halal certificate. ^13^ Meanwhile, entrepreneurs in other Indonesian cities such as the Greater Jakarta Area, ^14^ Malang City East Java Province, ^15^ and Surakarta City Central Java Province ^16^ did not register halal certification because they perceive the process as difficult and costly. These cases indicate MSME entrepreneurs’ lack of necessity for obtaining halal certificates.

In another case, most street vendors (“kaki lima”) in the nearby area of Universitas Islam Bandung (Unisba), which is located in Bandung, the capital of West Java Province, Indonesia, do not have halal certificates, although they serve thousands of Muslim students and employees daily. They are unconcerned about the halal status of the materials or the food they sold. They have limited knowledge of halal products, the procedures for obtaining halal certificates, and a lack of desire to obtain them. ^17^

According to our observations, the majority of MSE food producers in West Java Province, Indonesia, come from low- to middle-income families with limited educational opportunities. According to the Central Statistics Agency, 61.63% of UMK owners in West Java have the highest education in elementary school with an annual income ranging from 10 and 24 million rupiahs. ^18^ Many of them are solely concerned with producing and selling goods. They have had enough as long as they have sold it. As a result, it led to the assumption that halal certificates are not important. The problems are that the MSE food producers may be unaware that raw materials are being processed or that the processing method, how to store, and how to send products do not meet halal standards, resulting in a low intention to obtain a halal certificate. Hence, this study aims at measuring the level of halal awareness and the intention of MSE food producers in West Java Province, Indonesia to register halal certification.

This paper is organized as follows; the literature review, the proposed conceptualizing model, the research method, the results, the discussion of the results, the conclusions, and data availability.

### Literature review

Studies on halal awareness and intention to register halal certification have been carried out in the last decade. Giyanti and Indriastiningsih ^16^ hypothesized that awareness/intention to register a halal certificate is influenced by knowledge of halal, perception of benefits, and perception of procedures. The study results show that most SMEs food in Surakarta City, Central Java Province, Indonesia have a good knowledge of halal and agree that halal certification benefits their business. However, knowledge of halal does not significantly affect halal certification. Only the perception of the benefits of halal certification significantly influences the intention to obtain halal certification. They do not register halal certification due to a lack of understanding of the procedure for obtaining a halal certificate.

Abdul *et al*., ^19^ investigated the perception of halal certification among SME food entrepreneurs in Yogyakarta City, Indonesia. Food entrepreneurs who already have halal certificates report that the halal certification process is not tedious or stringent. They learn a lot about halal while going through the certification process. They also believe that halal certificates can increase market share and expand their business by instilling consumer trust in their products, i.e., providing a sense of security. Furthermore, halal-certified products are more competitive. On the other hand, SME food entrepreneurs who do not have halal certificates, suppose that the certification process is complicated and time-consuming. This was also stated by Viverita and Kusumastuti, ^14^ Giyanti and Indriastiningsih, ^16^ and Santosa *et al*. ^20^ It could be due to insufficient information about the process of obtaining a halal certificate and the benefits of having halal-certified products. ^14^ ^,^ ^19^ These studies indicate that intention to obtain halal certification is affected by the perception of the procedure.

According to Liba *et al*., ^9^ Elias *et al*., ^21^ and Masithoh *et al*., ^22^ awareness of halal is positively correlated with the intention to obtain halal certification. Meanwhile, Dinev and Hu, ^23^ ^–^ ^26^ Bachok *et al.*, ^24^ Lee and Shin, ^25^ Rezai *et al*., ^26^ state that customer awareness is a strong predictor of a customer’s intent to buy or select a product. These indicate that awareness influences intention.

Waluyo ^27^ presumes that religious understanding, profit motivation, and level of education influence the awareness of halal-certified food producers in Sleman and Bantul, Yogyakarta Special Region, Indonesia. The significance test results show that the variables of religious understanding and profit motivation have a significant effect on awareness of being certified halal.

### Halal awareness

The Law of Republic Indonesia No. 33/2014 describes halal products as those that conform to Islamic Sharia (principles). Carrions, blood, pigs, and/or halal animals (e.g., chicken, cow, goat, etc.) slaughtered in a manner inconsistent with Islamic Sharia are all considered non-halal (haram) materials. Furthermore, non-halal materials also include intoxicating plants or drinks, material that is harmful to one’s health, and microbes contaminated with non-halal materials. Halal encompasses substances ( *dzatihi*), the nature of the substances, processes, processing areas, processing instruments, product storage, product distribution, and serving. ^27^ Based on these explanations, this study defines halal as what is permissible for Muslims to eat, drink, and use under Islamic law.

Awareness is defined as the state of being aware: knowledge and understanding that something is happening. ^28^ According to the definition of halal used in this study and the definition of the word awareness in the dictionary, halal awareness is then conceptualized as a process of being aware of what is allowed for Muslims to eat, drink, and use. The level of halal awareness is influenced by religious beliefs, exposure, the role of halal certification through the halal logo/label, health-related reasons, ^29^ genders, marital status, ^30^ religious knowledge, and motivation to gain profit. ^27^

The religious knowledge of halal considers knowledge of the laws relating to what allowed Muslims to eat, drink, and use as described in the Quran and Hadith. All foods are generally permitted except for those derived from prohibited animals such as pigs, dogs, and carrion, as well as foods and beverages containing alcohol and other toxic or dangerous substances. Slaughter must be carried out following Sharia, to do so in the name of God. ^31^ Allah says in the Quran Surah (chapter) 2 (Al Baqarah) ayah (verse) 173 as follows ^32^:

*He has only forbidden to you dead animals, blood, the flesh of swine, and that which has been dedicated to other than Allah. But whoever is compelled (by necessity), without (willful) disobedience nor transgressing (the limits) then there is no sin on him. Indeed, Allah is Oft-Forgiving, and Most Merciful.*

Meanwhile, motivation to gain profit is defined as the food entrepreneur’s perception of the effect to be gained by producing halal food or having halal certificates and labels. The benefits of producing halal food include increasing market share and competitiveness, ^33^ business growth, ^1^ or business development. ^2^

### Attitude and intention to register halal certification

Several previous studies show that there is a positive relationship between attitude and intention to buy or choose a product. ^34^ ^–^ ^36^ According to the Planned Behavior Theory, the intention is determined by three independent factors, i.e., attitude toward behaviour, subjective norm, and perceived behavioural control. Supposing that there is a positive attitude that is supported by people around (as a subjective norm) and there is a perception of ease to perform the behaviour under consideration (as a behavioural control), then an individual’s intention to behave will be higher. ^37^

From an Islamic perspective, every Muslim must have an attitude to like and want to do a good job. In a broader context, attitude means to do good deeds due to Allah (God) loves those who do good as Allah (God) commands in Quran surah (chapter) Al-Baqarah ayah (verse) 195: ^32^

*And spend in the way of Allah and let not your own hands throw yourselves into destruction. And do good; indeed, Allah loves the good-doers.*

Attitude is also associated with two conditions i.e. good (‘ *mahmudah*’) and bad (‘ *mazmumah*’). ^38^ Hence, from an Islamic perspective and according to the Planned Behaviour Theory, if a Muslim produces halal foods as a do good (as an attitude toward a behaviour), supported by an awareness that Allah promises to love the good-doers (as a subjective norm), and there is a perception of ease of the procedure to obtain halal certification (as a behavioural control), then the individual’s intention to register halal certification will be higher.

The procedure to obtain the halal certificate is conceptualized as the food entrepreneur’s perception of the steps that must be taken to obtain a halal certificate and label. Standards for gaining halal certification in Indonesia are explained in the Law of Republic Indonesia No. 33/2014 concerning the guarantees of halal products. ^10^

## Conceptualizing awareness and intention to register halal certification

### Identification of variables

According to Waluyo, ^27^ motivation to obtain halal-certified is significantly influenced by religious understanding and motivation to gain profit, because SME entrepreneurs generally agree that Halal Food Certification provided benefits. ^16^ However, the procedure for obtaining halal certificates is relatively complex, thereby reducing the intention of SMEs to register halal certification. Referring to the Planned Behavior Theory ^37^, the intention to register for halal certificates is influenced by attitude to produce halal products, supported by halal awareness, and perception of the ease of procedure to obtain halal certificates.

Referring to Giyanti and Indriastiningsih, ^16^ Waluyo, ^27^ and Ajzen, ^37^ this study identifies that the variables are knowledge of halal (KH), perception of benefits (PB), perception of procedures (PP), halal awareness (HA), attitude to produce halal foods (AHC) and intention to register a halal certificate. Measurement items of each variable are then compiled from the previous studies. ^10^ ^,^ ^16^ ^,^ ^27^ ^,^ ^29^ ^,^ ^30^ ^,^ ^33^ ^,^ ^39^

### Conceptual model

**Figure 1.**
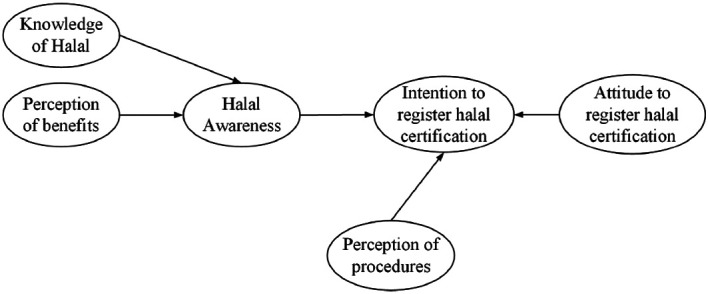
Conceptual model of awareness and intention to register halal certification.

Referring to Giyanti and Indriastiningsih, ^16^ and Waluyo ^27^ this study considers that halal awareness is influenced by knowledge of halal and MSEs’ perception of the benefit of the halal certificate. Referring to the Planned Behavior Theory, halal awareness (as a subjective norm), attitude to produce halal foods (as a positive attitude), and MSEs’ perception of ease of procedures (as a behavioural control) will encourage the intention for registering halal certification. The relationship of these variables represents the conceptual model of halal awareness and intention toward halal certification (see Figure 1). We introduce a halal awareness between knowledge of halal or perception of benefit and intention to register halal certification.

As can be seen in Figure 1, halal awareness is a dependent variable, while knowledge of halal and perception of benefits are independent variables. Furthermore, halal awareness, perception of procedures, and attitude to produce halal foods are independent variables, while the intention to register halal certification is a dependent variable.

### Hypothesis

The conceptual model ( Figure 1) shows that the level of halal awareness is influenced by the knowledge of halal which may include MSEs’ understanding of the types of non-halal food as mentioned in Law of Republic Indonesia No. 33/2014. ^10^ Therefore, we hypothesize that:

*H1: The knowledge of halal/non-halal levels (KH) positively affects Halal Awareness (HA) of the MSE food producers.*

Halal certificates and halal logos are perceived to have benefits in increasing consumer confidence and competing with other producers, ^30^ using as a promotional tool. ^16^ Hence, halal certificates are expected to improve the MSEs’ performance. Based on this point of view, we hypothesize that:

*H2: Perception of benefits (PB) positively affects halal awareness (HA).*

Halal awareness is measured by awareness of the importance of using halal materials in producing halal products, ^9^ and perceiving the benefits to be gained despite the process being very strict. ^30^

Consumer awareness is a strong predictor of their intention to consume/purchase foods. ^24^ ^,^ ^26^ In this study, halal awareness is expected to influence on intention to register halal certification. Hence, we hypothesize that:

*H3: Halal awareness (HA) positively affects the Intention to register Halal certification (IHC).*

MSE entrepreneurs perceive that the procedure to achieve halal certification is complex due to the lack of information from respondents regarding halal certification procedures. ^16^ This will negatively influence the intention of producers to register halal certification. In the light of this, we hypothesize that:

*H4: The MSEs’ perception of ease of the procedures (PP) positively affects the Intention to register halal certification (IHC).*

A positive relationship between attitude and intention has been shown by Rezai and Teng, ^26^ Jaafar *et al.*, ^34^ Yang *et al*. ^36^ These show that attitude influences intention. Hence, we hypothesize that:

*H5: Attitude to produce halal foods (AHC) positively affects the Intention to register halal certification (IHC).*

## Methods

### Study design and participants

This study adopts a quantitative method to evaluate the hypothesis, i.e., analyze the data using descriptive statistics. This study has followed the STROBE guidelines/checklist for cross-sectional research.

### Measurement items

The measurement items for each variable are identified in the following explanation. All variables, indicators, and measurement items are described in Table 1.

(i) Knowledge of halal (KH)

**Table 1.** Variables, indicators, and measurement items of awareness and intention to register halal certification.

Variables	Indicators	Measurement items	References
Knowledge of Halal (KH)	KH1	Halal animals are slaughtered not following Islamic Sharia is non-halal ( *haram*)	^16^
	KH2	Products containing alcohol used in the production process are non-halal ( *haram*)	^27^
	KH3	Pork and its derivation used in the production process are non-halal ( *haram*)	
	KH4	Equipment used to produce halal food must be kept separate from equipment used to produce non-halal food	
Perception of Benefits (PB)	PB1	Halal certificate can be used as a promotional tool	^16^
	PB2	The ownership of a Halal certificate increases consumer trust in MSE products	^30^
	PB3	The Halal certificate contributes to the development of MSE performance	
	PB4	The halal certificate will make MSEs more competitive	
Perception of Procedures (PP)	PP1	The MSE has sufficient information on the halal certification process	^29^
	PP2	Halal Certification is a relatively simple process	^16^
	PP3	The cost of maintaining halal certification is cheap for MSE	
	PP4	The time of obtaining halal certification is relatively quick	
Halal Awareness (HA)	HA1	The MSE is aware of the importance of producing halal food	^10^
	HA2	The MSE is aware of the importance of using halal raw materials	^30^
	HA3	The MSE is aware of the importance of a halal certificate	
	HA4	The MSE is aware of the rigorous certification process	
Attitude to produce halal foods (AHC)	AHC1	The MSE is always concerned about a product’s halal issue	^29^
	AHC2	As a food producer, the MSE is always concerned that its customers purchase products that follow Islamic Sharia	
	AHC3	The MSE is always concerned with producing halal products	
	AHC4	The MSE ensures that the raw materials are halal at all times	^10^
Intention to Register Halal Certification (IHC)	IHC1	Although the MSE ensured that halal materials were used, the MSE is still in charge of halal certification	^33^ ^,^ ^39^
	IHC2	The MSE must try to comply with halal quality standards to obtain halal certification	
	IHC4	The MSE will register the products of MSEs to obtain halal certification	
	IHC3	The MSE will apply the Halal assurance system in their business	^10^

According to the previously defined, knowledge of halal in this study only includes knowledge about halal/non-halal (haram) materials according to the Quran and Hadith, as well as knowledge about the separation of equipment used to process halal/non-halal (haram) materials. The measurement items for the knowledge halal variable were adapted from Giyanti and Indriastiningsih ^16^ and Waluyo. ^27^ The survey section includes four items, such as knowledge of; slaughtering methods (KH1), haram material and products (KH2 and KH3), and processing equipment (KH4).

(ii) Perception of benefits (PB)

The measurement items for the perception of benefits variable were adapted from Giyanti and Indriastiningsih, ^16^ and Abdul. ^30^ These items asked about respondents’ perception of the benefits they would get if they had a halal certificate, such as a promotional tool (PB1), more convincing consumers to buy (PB2), improving business performance (PB3), and more competitive (PB4).

(iii) Perception of procedures (PP)

In this study, food entrepreneurs’ perception of the procedure for obtaining halal certificates in Indonesia involves the availability of information about the certification process and the fact that the certification process is simple, inexpensive, and quick. The measurement items for the perception of the procedure variable were asked about the respondent’s perception of the procedure for obtaining halal certificates in Indonesia, such as the existence of information about the certification process (PP1), the certification process is easy (PP2), cheap (PP3), and fast (PP4). These items were adapted from. ^16^ ^,^ ^29^

(iv) Halal awareness (HA)

The Law of Republic Indonesia No. 33/2014 defines the halalness of products, including material supply, processing, storage, packaging, distribution, sales, and product presentation. According to our observations, most MSE food producers in West Java only produce one type of food, either sold directly at their “warung” (a little shop) or entrusted to someone else’s “warung”. Thus, they buy raw materials, process them into finished goods, and then store or ship one kind of product only. Hence, this study limits the scope of halal awareness as the awareness of using halal materials and processing them in a halal manner.

The measurement items for the halal awareness variable were asked about the awareness of respondents about the importance of producing halal food (HA1), using halal raw materials (HA2), the importance of having a halal certificate (HA3), and the rigorous of the halal certification process (HA4). These adapted from Abdul22 and Law of Republic Indonesia No 33/2014. ^10^

(v) Attitude to produce halal foods (AHC)

This study conceptualizes attitude as thinking about producing a halal product that includes a focus on halal issues, a guarantee of selling halal products, halal product attention, and checking to use of halal raw materials. Hence, the measurement items for attitude to produce halal foods variable include respondents’ attitudes to always pay attention to halal issues (AHC1), ensure consumers buy halal products (AHC2), pay attention to halal products (AHC3), and use halal materials (AHC4). These items were adapted from Ambali and Bakar21 and Menteri Hukum dan HAM (Minister of Law and Human Rights-English). ^10^

(vi) Intention to register halal certification

The intention is conceptualized as an act of registering the product to obtain a halal certificate. The measurement items for the variable of intention to register halal certification were adapted from Law of Republic Indonesia No 33/2014, ^10^ Abdul *et al*., ^33^ and Ngah *et al*. ^39^ These items include respondents’ intention to be responsive to the certification process (IHC1), strive to meet halal quality standards (IHC2), immediately implement halal assurance system (IHC3), and immediately register halal certification (IHC4).

### Population and sample

The study was carried out in West Java Province, Indonesia. The population is the MSE food producers listed in the Central Bureau of Statistics of West Java, such as producers of cassava chips, shredded catfish, “bagelen” cakes, chocolate “rangginang”, candied vegetables, market snacks, and so on. A copy of the list of West Java MSEs registered in the Office of Cooperatives and Small Businesses in West Java Province obtained from the Central Bureau of Statistics of West Java can be found in Underlying Data. ^40^

The Categorization of business scale refers to the “Undang-Undang Republik Indonesia Nomor 20/2008” (Law of the Republic of Indonesia Number 20/2008). ^41^ According to that law, the criteria for micro-enterprises are as follows:

a. having a maximum net asset of Rp. 50,000,000.00 (fifty million rupiahs), excluding land and buildings for business premises; or
b. having a maximum annual sales turnover of IDR 300,000,000.00. (three hundred million rupiahs).

Whereas in article 6 paragraph 2 it is stated that the criteria for small businesses are as follows:

a. having a net asset of more than IDR 50,000,000.00 (fifty million rupiahs) up to a maximum of IDR 500,000,000.00 (five hundred million rupiahs) excluding land and buildings for business premises; or
b. having an annual sales turnover of more than IDR 300,000,000.00 (three hundred million rupiahs) up to IDR 2,500,000,000.00 (two billion five hundred million rupiahs).

Table 2 shows the categorization of the business scale.

MSE food producers chosen as respondents include those who meet the legal criteria. Furthermore, the purpose of this study is to determine the awareness level of MSE food producers regarding halal certification. Hence, the determined criteria for respondents were as follows:

1) a food producer of micro and small-scale with annual sales turnover of fewer than 2,500 million Rupiahs and net assets of fewer than 500 million Rupiahs;
2) have an ongoing business, and
3) no halal certificate.

**Table 2.** Category of micro, small and medium business in Indonesia according to Law of the Republic of Indonesia No. 20/2008. ^40^

Category	Net assets (in million Rp.)	Annual sales turnover (in million Rp.)
Micro	50 (max)	300 (max)
Small	50 – 500	300 – 2,500

The purposive sampling technique was used in this study. The total number of food producers of MSE listed in the Central Bureau of Statistics of West Java was estimated to be around 2300 people. We worked with “Sahabat UMKM Jawa Barat” (West Java MSME association-English), a local association of micro, small, and medium-scale entrepreneurs who are engaged in a variety of fields such as culinary, fashion, crafts, and other businesses or industries. The local association has over 1,000 members, with 68 percent of them being food and beverage entrepreneurs, or approximately 680 entrepreneurs. This study’s sample consists of food producers who meet the determined criteria among the 680 entrepreneurs.

We received written permission from the Chairman of the West Java MSME association to contact his members for data collection. The written consent to participate from the Chairman of the West Java MSME Community was gained according to document number: 015/SKIP/IV/2020. To guarantee that there is no conflict of interest in this study, survey responses are kept anonymous.

### Ethical consideration

The Ethical Licensing Committee of the Islamic University of Bandung approved this study by Protocol number: 495/B.04/Bak-k/XII/2019. We provided all respondents with a consent statement after consultation. In the questionnaire, there is a statement that by filling out the questionnaire the respondents gave their consent to participate. Respondents gave their consent to take part when they filled out the questionnaire. Respondents had given their consent truly and without coercion. Furthermore, to protect respondents’ rights and privacy, all forms of data obtained will be kept confidential.

### Data collection

A questionnaire is chosen as the research instrument. The questionnaires were re-translated from English to Indonesian, except for those references that were already in Indonesian. Each variable is made up of measurement items which are scored on a Likert scale of 1 (strongly disagree) to 5 (strongly agree).

The West Java MSME association, which has over 1,000 members with 680 food and beverages entrepreneurs, has set up several WhatsApp groups to help them communicate with one another. Some groups exist due to the limited number of members who can join a single WhatsApp group. We collect data through these WhatsApp groups. A Google Form with a cover letter and a set of questionnaires were sent out electronically to the potential respondents who are members of the West Java MSME association WhatsApp groups. without separating food and non-food entrepreneurs. This method was chosen because of the COVID-19 pandemic outbreak. In addition, the designed questionnaires could be collected without conducting direct visits to the respondents. The respondents could not participate in the survey unless they gave their written consent. Data were collected from March to May 2020. A copy of the distributed questionnaires can be found in Extended Data. ^42^

The questionnaire was pretested with a small sample of members of the West Java MSME association before being distributed to the actual respondents. Based on pretest feedback, the wording of some items was refined and modified to guarantee that the validity and reliability of each variable meet the required standard. The question items were scored on a Likert scale of 1 (strongly disagree) to 5 (strongly agree). The follow-up of this plan is described later in the data preparation section.

The purposive sampling method is used in the following manner.

1) Distribute questionnaires.
2) Wait for responses to the distributed questionnaire.
3) Collect data until the sample count is adequate.
4) Screen participants based on the criteria specified.

### Structural model analysis

Data are analyzed with descriptive statistics to provide a description of the respondents’ profile, and to describe the results of the assessment of the level of knowledge of halal, perception of benefits, halal awareness, perception of procedure, attitude, and intention to register halal certificates. The research analysis is intended to assess the model and objectively describe the hypotheses.

The adopted theoretical models are evaluated using Partial Least Square – Structural Equation Model (PLS-SEM) with a path model because this study is exploratory research to predict certain constructs by focusing on explaining the variance in the dependent variables when examining the model. ^43^ A Smart-PLS software is chosen for data processing.

There are two elements in the PLS-SEM with path models: the outer model and the inner model. The outer model (also known as the measurement model) describes the relationships between latent variables and their indicators, whereas the inner model (also known as the structural model) depicts the relationships between latent variables. ^43^ ^,^ ^44^

The assessment of the PLS-SEM model begins with the measurement models (outer model) by evaluating the quality of the reflective or formative measurement models. The reflective measurement models are estimated by assessing the construct measures’ reliability and validity. Composite reliability (as a means of assessing internal consistency reliability), convergent validity, and discriminant validity are among the specific measures. Formative measures are evaluated for convergent validity, indicator weight significance and relevance, and the presence of collinearity among indicators. ^43^ ^,^ ^44^

Following confirmation that the construct measures are reliable and valid, the structural model (inner model) results are evaluated. This entails investigating the model’s predictive abilities for theory testing, as well as the relationships between the constructs. The PLS-SEM model fit is evaluated using standardized root mean square residual (SRMR), root mean square residual covariance (RMS _theta_), or the exact fit test to determine how well it predicts endogenous variables/constructs. ^43^

The first step in evaluating the PLS-SEM results for the structural model is to look at the significance and relevance of the coefficients. The bootstrapping routine and examining *t* values, *p* values, or bootstrapping confidence intervals are required to test the significance. Despite this, the bootstrapping confidence interval is less common. ^43^ Following that, the relative sizes of path coefficients, total effects, *f* ^2^ effect size, *Q* ^2^ effect size, and *q* ^2^ effect size can be compared. By interpreting these findings, the key constructs with the greatest relevance to explaining the endogenous latent variable(s) in the structural model can be identified. ^43^ ^–^ ^45^ Concisely, the systematic evaluation of PLS-SEM output is shown in Table 3.

**Table 3.** Systematic evaluation of PLS-SEM output. ^43^ ^–^ ^45^

Criteria	Assessed value	Acceptable value
**I. Evaluation of the reflective measurement model**		
Convergent validity	Loading	>0.7
	Indicator Reliability	>0.5
	AVE	>0.5
Internal Consistency Reliability	CR	>0.6
	Cronbach Alpha	>0.6
Discriminant Validity	Fornell-Larcker	The square root of each construct’s AVE should be higher than the correlations among the latent variables
	Cross-loading	An indicator has a lower correlation with another latent variable than with its respective latent variable
**II. Evaluation of the structural model**		
Model Fit	SRMR	<0.08
	RMS _theta_	<0.12
*R* ^2^ of endogenous latent variables	*R* ^2^ values of 0.75, 0.50, or 0.25 for endogenous latent variables are considered substantial, moderate, or weak, respectively	
Estimates of path coefficient	For one-tailed tests, the critical values are 1.28 (significance level = 10%), 1.65 (significance level = 5%), and 2.33 (significance level = 1%) To conclude that the relationship under consideration is significant at a 5% level, the p-value must be less than 0.05 when assuming a significance level of 5%	
Effect size *f* ^2^	Small, medium and large effects of the exogenous latent variable are represented by *f* ^2^ values of 0.02, 0.15, and 0.35, respectively	
Predictive Relevance *Q* ^2^	*Q* ^2^ values greater than zero for a specific reflective endogenous latent variable indicate that the path model is predictive of a specific dependent construct	

### Sample size

Barclay, Higgins, and Thompson (1995) in Hair *et al*. ^43^ explain the Ten Times Rule in determining the number of PLS-SEM samples, which states that the sample size must be greater than (1) ten times the greatest number of formative indicators used to measure a single construct, or (2) ten times the greatest number of structural paths directed at a specific construct in the structural model. In other words, the minimum sample size is equal to 10 times the maximum number of arrows in the PLS path model pointing to the latent variable. ^43^

In this study, the IHC variable is the latent variable with the maximum number of arrows, i.e., 3 (see Figure 1). As a result of the Ten Times Rule, 3.10 = 30 represents the bare minimum of observations required to estimate the PLS path model depicted in Figure 1. In terms of Cohen’s (1992) recommendation for multiple OLS regression analysis, or running a power analysis using the G*Power program, as cited in Hair, *et al*., ^43^ 33 observations are required to detect an *R* ^2^ value of about 0.25, assuming a statistical power of 80% and significance level of 5%.

## Results

### Participants

The questionnaires were distributed to the members of the West Java MSME association’s WhatsApp groups. The questionnaires were returned by 376 respondents, including food and non-food entrepreneurs with micro, small, and medium-scale businesses. All respondents who returned the questionnaires were then selected based on the determined criteria with the following stages:

1 divide them into two groups: those with medium-sized businesses and those with small or micro-scale businesses,
2 separate those with micro or small businesses from those with medium-scale businesses
3 choose the micro or small-scale businesses with food business product types.
4 select the micro or small-scale food producers who do not have halal certificates

Respondents who were chosen up to the fourth stage were referred to as “selected”, while the others as “non-selected”. There were 137 selected respondents and 239 non-selected respondents as a result of the selection.

We examined the responses of the selected and non-selected respondents to see if there was any possibility of non-response bias. Lindner *et al*., ^46^ proposed three methods for investigating non-response bias: (1) comparing early to late respondents, (2) using “days to respond” as a regression variable, and (3) comparing respondents to non-respondents. This study adopted the third proposed method of Lindner *et al.*, ^46^ by comparing selected and non-selected respondents. We examine 20% of both selected and non-selected respondents. That is, 27 respondents (20% of 137) were taken from the selected group, while 47 respondents (20% of 239) were taken from the non-selected group.

The independent samples *t*-test was used to compare the responses of the two groups due to the difference in the number of respondents examined.

The findings show that there is no bias when none of the respondents in the two groups tested registered their products to obtain a halal certificate. When the non-selected respondents were a mix of those who had not yet registered and those who had already obtained halal certificates, two indicators were discovered to be biassed in the perception of procedure variable (PP), namely PP2 and PP4 indicators, or in the perception of benefit variable (PB), namely PB4 indicator. This indicates that respondents who have obtained halal certificates have different perceptions than those who have not, particularly regarding perceptions of simplicity (PP2) and the length of time to obtain a halal certificate (PB4), as well as benefits such as increased competitiveness from having a halal certificate.

Table 4 displays the percentage of respondents for each indicator where 98.5% of respondents have Islam as their religion (Muslim), 67.9% are female, and 38% are 26 years old or older. In terms of business size, 89% of respondents are micro-scale entrepreneurs. Non-Muslim respondents are involved because producing halal food regardless of the religion of the food producers.

**Table 4.** Respondent’s profile.

Indicator		Quantity	Percentage
Religion	Islam	135	98.5%
	Catholic	1	1.5%
	Protestant	1	1.0%
Gender	Male	44	32.1%
	Female	93	67.9%
Age	<17 years	1	0.7%
	17-20 years	7	5.1%
	21-25 years	10	7.3%
	26-40 years	52	38%
	>40 years	67	48.9%
Business scale	Micro	122	89.0%
	Small	15	11.0%

### Descriptive statistics of the research variables

Table 5 shows the descriptive statistics for each measurement indicator. The knowledge of halal (KH) has a high average perception value.

**Table 5.** Descriptive statistics measurement indicators.

Indicator	Minimum	Maximum	Mean	Total mean	Deviation standard
KH1	1	5	4.650	4.621	0.885
KH2	1	5	4.460		1.025
KH3	1	5	4.723		0.886
KH4	1	5	4.650		0.842
PB1	1	5	4.591	4.584	0.859
PB2	1	5	4.657		0.850
PB3	1	5	4.577		0.926
PB4	1	5	4.511		0.982
HA1	1	5	4.650	4.559	0.842
HA2	1	5	4.708		0.812
HA3	1	5	4.628		0.854
HA4	1	5	4.248		1.009
PP1	1	5	3.401	3.159	1.156
PP2	1	5	3.153		1.177
PP3	1	5	3.052		1.173
PP4	1	5	3.029		1.214
IHC1	1	5	4.504	4.544	0.855
IHC2	1	5	4.562		0.853
IHC3	1	5	4.577		0.869
IHC4	1	5	4.533		0.905
AHC1	1	5	4.526	4.591	0.889
AHC2	1	5	4.628		0.863
AHC3	1	5	4.699		0.758
AHC4	1	5	4.511		0.919

### Data preparation

A copy of the dataset of the questionnaire result can be found in Underlying Data. ^47^ There are no missing values, invalid observations, or outliers in this data set, which has a sample size of 137.

Initially, the questionnaires were first to be pretested in a small sample of MSE food producers in West Java, Indonesia, to assess the research instrument’s validity and reliability. The questionnaires are planned to be distributed in person starting in March 2020. However, we were unable to meet with the food producer due to the social distancing caused by the COVID-19 outbreak. Finally, we decided to distribute the electronic questionnaire Google Forms in May 2020 to the West Java MSME association’s WhatsApp groups. In just a few days, the data collected reached 137 respondents who met the predetermined criteria, so all collected data were subjected to validity and reliability tests.

### Measurement model (Outer model) analysis

Based on the conceptual model shown in Figure 1 and the measurement items in Table 1, the structural model involves the following 2 models:

1. Model of the influence of KH and PB on HA, where HA is an endogenous latent variable, while KH and PB are exogenous latent variables.
2. The influence model of HA, PP, and AHC on IHC, where IHC is an endogenous latent variable, while HA, PP, and AHC are exogenous latent variables.

**Figure 2.**
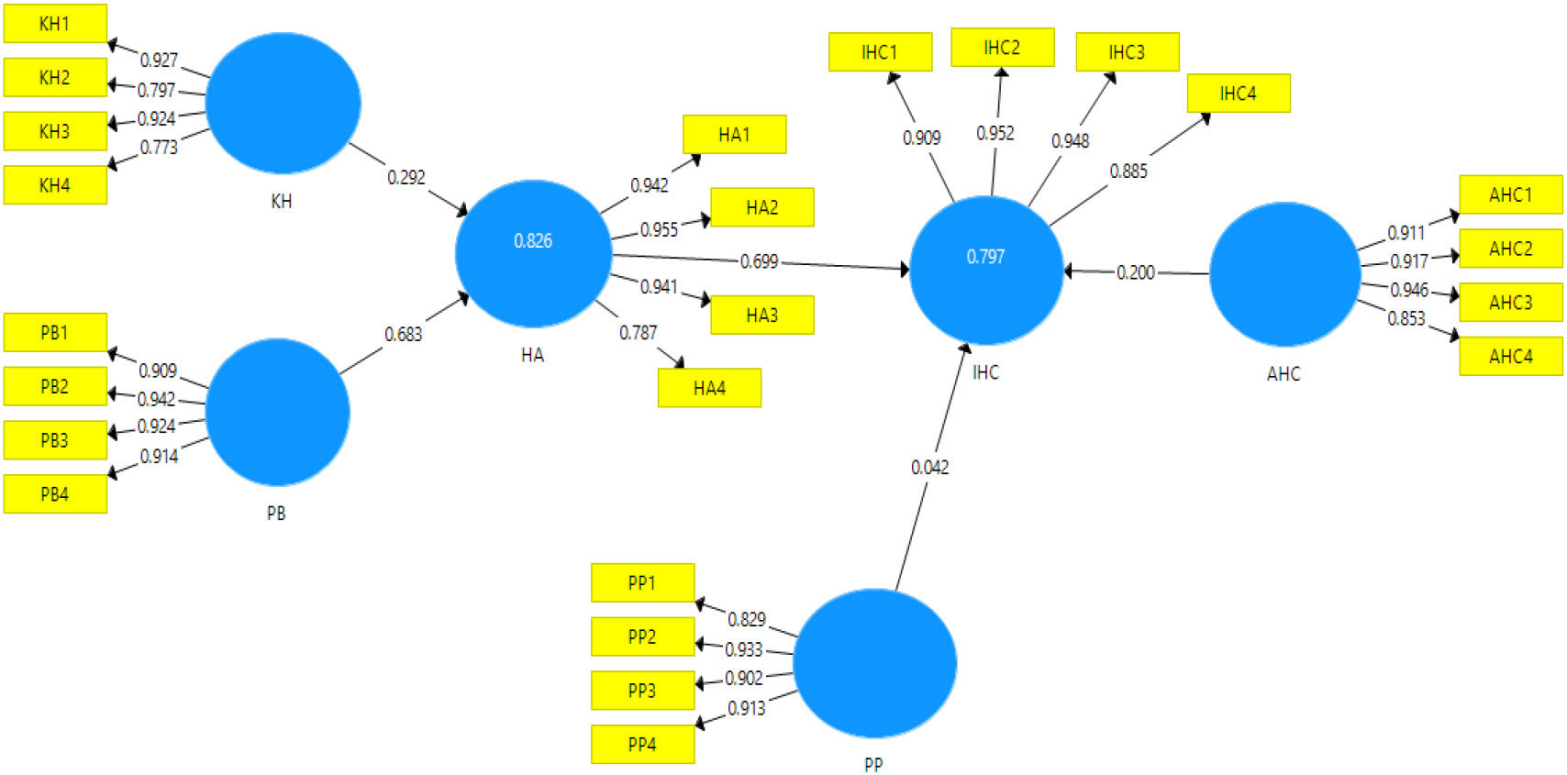
PLS-SEM result of the first path analysis.

The constructs or latent variables in this structural model are KH, PB, HA, PP, AHC, and IHC. Each latent variable is made up of some indicators that are highly correlated and interchangeable, allowing them to be reflective. Hence, the causality flow is going from the construct to the indicators. It means that any changes in the construct are expected to be reflected in all of its indicators (see Figure 2). Reflective measurement models should be evaluated for their reliability and validity. The path analysis for the proposed model is obtained by running Smart PLS-SEM software, as shown in Figure 2.

### Outer model loading

The correlation between the latent variable and the indicators in its outer model is evaluated by an outer loading. The outer loading of the first path model is shown in Table 6 where the outer loading of all indicators is greater than 0.7, indicating that it meets the convergent validity criteria. However, when the discriminant validity was examined, it was discovered that the correlation between AHC-HA is higher than AHC-AHC, while the correlation between IHC-HA is higher than HA-HA (see Table 7), indicating that the path model does not meet the Fornell-Larcker criterion.

**Table 6.** Outer loading of the first path model

	AHC	HA	IHC	KH	PB	PP
**AHC1**	0.933					
**AHC2**	0.943					
**AHC3**	0.915					
**AHC4**	0.904					
**HA1**		0.925				
**HA3**		0.958				
**HA4**		0.835				
**IHC1**			0.946			
**IHC3**			0.936			
**IHC4**			0.952			
**KH1**				0.901		
**KH2**				0.849		
**KH3**				0.910		
**KH4**				0.838		
**PB1**					0.941	
**PB2**					0.959	
**PB3**					0.945	
**PB4**					0.936	
**PP1**						0.822
**PP2**						0.921
**PP3**						0.870
**PP4**						0.871

**Table 7.** Discriminant validity construct of the first path model.

	AHC	HA	IHC	KH	PB	PP
**AHC**	0.924					
**HA**	0.925	0.916				
**IHC**	0.883	0.917	0.945			
**KH**	0.729	0.735	0.687	0.875		
**PB**	0.849	0.911	0.897	0.696	0.945	
**PP**	0.304	0.249	0.304	0.269	0.303	0.872

Hence, the model’s feasibility must be reconsidered ^45^ by analyzing the multicollinearity to determine whether any indicators should be merged into one or eliminated. ^43^ ^,^ ^44^ Collinearity is assessed by calculating a variance inflation factor (VIF) for each indicator and comparing these VIFs to a threshold. The VIF threshold values of 10, 5, and 3.3 are commonly recommended for collinearity, which means that a VIF equal to or greater than the threshold value indicates a potential collinearity problem. ^48^ As a result, the corresponding indicators should be considered to be removed. ^43^

Table 8 displays the VIF value of each indicator of the first path model. As can be seen, it was discovered that the HA2 indicator had a VIF value greater than 10, so the HA2 indicator was considered to be discarded. After the HA2 indicator is removed, the processing is performed on the second path model, which does not include the HA2 indicator. Based on the discriminant validity analysis, it was discovered that the correlation between IHC-HA is higher than HA-HA. Hence, the VIF value should be checked again. The calculation result shows that there are no VIF values greater than 10, but the IHC2 indicator has close to 10 values of 9.02. To meet the Fornell-Larcker criterion, the IHC2 indicator is being considered for removal. The third path model is then constructed without the use of the HA2 and IHC2 indicators. When the discriminant validity of the third path model was examined, it was discovered that all latent variables met the Fornell-Larcker criteria. Consequently, the final path model for the problem discussed in this study is the third path model.

**Table 8.** Outer VIF values of the first path model.

Indicators	VIF	Indicators	VIF	Indicators	VIF
**AHC1**	4.467	**IHC1**	5.711	**PB1**	5.647
**AHC2**	5.226	**IHC2**	9.020	**PB2**	7.203
**AHC3**	3.873	**IHC3**	3.857	**PB3**	5.527
**AHC4**	3.355	**IHC4**	7.774	**PB4**	5.105
**HA1**	8.685	**KH1**	2.986	**PP1**	1.745
**HA2**	11.475	**KH2**	2.678	**PP2**	3.732
**HA3**	6.741	**KH3**	3.449	**PP3**	3.598
**HA4**	2.006	**KH4**	2.046	**PP4**	4.207

### The assessment of PLS-SEM output

In this study, both validity and reliability tests are carried out to measure the goodness of the shared questionnaires. Validity is a test of how well the developed instrument measures the particular construct being measured, while reliability is a test of how the developed instrument consistently measures the construct being measured. ^49^ The assessment’s steps are as follows:

(i) Checking to construct reliability and validity

According to the Systematic evaluation of PLS-SEM output described in Table 3, the findings of the reflective measurement model evaluation are summarized in Table 9. As can be seen, all items’ loadings exceed 0,7, the AVEs for the indicators are within the range of 0.760 and 0.893, all CR values are higher than 0.6, and the Cronbach Alpha’s are within the range of 0.892 and 0.960. Therefore, all model evaluation criteria have been met, indicating that the instruments are reliable and valid.

(ii) Checking Discriminant validity

The square root of the AVE of each construct should be greater than its highest correlation with any other construct, according to the Fornell-Larcker criterion. ^43^ Table 10 shows that the average variance extracted by the indicators measuring that construct is less than the squared correlations for that construct. To put it another way, the measurement model represents adequate discriminant validity.

Another method to determine discriminant validity is assessed by looking at loading and cross-loading to identify problem items if there are any. The validity test using cross-loading is patterned in that the main loading factor originating from its construct is greater than the correlation value built from these variables on other constructs. ^43^ Table 11 presents an evaluation of validity based on the value of the main loading factor to the value of cross-loading factors with other constructs. As shown in Table 11, the value of the main loading factor of each construct is higher than the value of the loading factor outside of the main loading factor, so it can be concluded that all constructs are declared valid. For example, the loading factor of indicator AHC1 in the AHC construct is highest than its loading factor in HA, IHC, KH, PB, and PP.

(iii) Model fit

This study uses the standardized root means square (SMSR) to assess model fit. The SMSR is 0.067, less than 0.08, indicating that the model meets the model fit criteria. Furthermore, the data can be used to estimate the model.

(iv) Hypothesis testing by checking structural path significance

**Figure 3.**
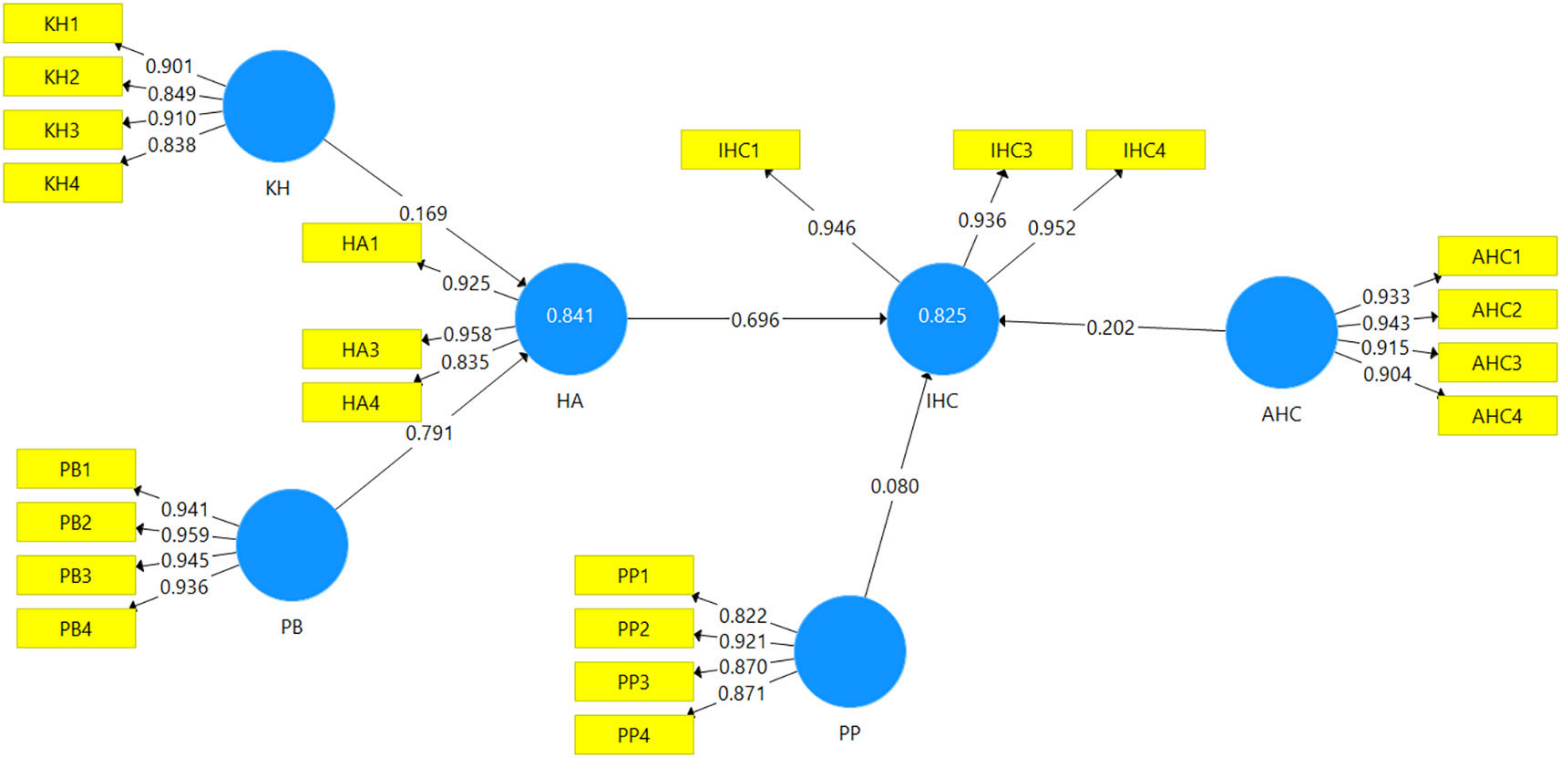
Result of the final path analysis.

Significance testing of both the inner and outer model in SmartPLS uses bootstrapping procedure to give approximate *t*-values. In this study, the hypotheses are represented with a positive path coefficient, so that the *t*-value is determined by the one-tailed *t*-test. By using a significance level of 5%, the path coefficient will be significant if the *t*-value is larger than 1.65. ^50^ Figure 3 shows the path analysis result of halal awareness and the intention to register halal certification using Smart PLS software, while Table 12 represents the hypothesis testing results of each path.

**Table 9.** Result summary for the reflective measurement model.

Variables	Indicator	Convergent validity			Internal consistency reliability	
		Loading factor	Indicator reliability (i.e. loading ^2^)	AVE	CR	Cronbach Alpha
		>0.7	>0.5	>0.5	>0.6	>0.6
KH	KH1	0.901	0.871	0.765	0.929	0.898
	KH2	0.849	0.889			
	KH3	0.910	0.836			
	KH4	0.838	0.818			
PB	PB1	0.941	0.856	0.893	0.971	0.960
	PB2	0.959	0.917			
	PB3	0.945	0.698			
	PB4	0.936	0.894			
HA	HA1	0.925	0.876	0.824	0.933	0.892
	HA3	0.958	0.906			
	HA4	0.835	0.811			
PP	PP1	0.822	0.720	0.760	0.927	0.897
	PP2	0.921	0.828			
	PP3	0.870	0.701			
	PP4	0.871	0.886			
IHC	IHC1	0.946	0.919	0.892	0.961	0.939
	IHC3	0.936	0.893			
	IHC4	0.952	0.876			
AHC	AHC1	0.933	0.676	0.854	0.959	0.943
	AHC2	0.943	0.848			
	AHC3	0.915	0.757			
	AHC4	0.904	0.758			

**Table 10.** Discriminant validity constructs the Fornell-Larcker criterion.

	AHC	HA	IHC	KH	PB	PP
**AHC**	0.924					
**HA**	0.912	0.908				
**IHC**	0.861	0.900	0.944			
**KH**	0.729	0.720	0.674	0.875		
**PB**	0.848	0.909	0.890	0.696	0.945	
**PP**	0.304	0.246	0.313	0.269	0.302	0.872

**Table 11.** Cross-loading for construct validity.

	AHC	HA	IHC	KH	PB	PP
**AHC1**	0.933	0.846	0.815	0.706	0.770	0.335
**AHC2**	0.943	0.869	0.820	0.734	0.836	0.266
**AHC3**	0.915	0.855	0.774	0.628	0.799	0.235
**AHC4**	0.904	0.802	0.773	0.620	0.728	0.287
**HA1**	0.873	0.925	0.823	0.719	0.841	0.192
**HA3**	0.873	0.958	0.907	0.698	0.906	0.256
**HA4**	0.728	0.835	0.706	0.528	0.714	0.220
**IHC1**	0.812	0.839	0.946	0.600	0.831	0.337
**IHC3**	0.784	0.824	0.936	0.613	0.820	0.296
**IHC4**	0.843	0.886	0.952	0.693	0.868	0.255
**KH1**	0.641	0.635	0.601	0.901	0.608	0.225
**KH2**	0.569	0.551	0.530	0.849	0.513	0.253
**KH3**	0.611	0.630	0.575	0.910	0.615	0.198
**KH4**	0.711	0.686	0.638	0.838	0.679	0.266
**PB1**	0.827	0.866	0.850	0.702	0.941	0.297
**PB2**	0.861	0.890	0.879	0.684	0.959	0.283
**PB3**	0.782	0.869	0.837	0.619	0.945	0.280
**PB4**	0.730	0.808	0.794	0.622	0.936	0.284
**PP1**	0.320	0.276	0.329	0.284	0.321	0.822
**PP2**	0.258	0.203	0.284	0.223	0.264	0.921
**PP3**	0.268	0.213	0.253	0.218	0.259	0.870
**PP4**	0.154	0.103	0.158	0.177	0.146	0.871

**Table 12.** Path coefficients and hypothesis testing results.

Hypothesis	Relationship	Coefficient	*t*-value	*p*-value	Remark ( *p* < 0.05)
H1	KH-HA	0.169	1.944	0.026	Significant
H2	PB-HA	0.791	9.062	0.000	Significant
H3	HA-IHC	0.696	5.824	0.000	Significant
H4	PP-IHC	0.080	1.633	0.052	Not significant
H5	AHC-IHC	0.202	1.586	0.057	Not significant

The path analysis is addressed to ascertain the hypotheses put forward. As can be seen in Figure 3, the *R* ^2^ value of 0.825 for IHC indicates that 82.5 % of the variance in IHC can be explained by HA, PP, and AHC. In addition, Figure 3 also shows that HA, PP, and AHC are positively related to IHC among MSE entrepreneurs with *β* = 0.696, 0.080, and 0.202, respectively. According to the *t*-value of the path coefficients, HA has a significant impact on IHC, while AHC and PP do not have a significant impact on IHC. Besides, the *R* ^2^ value of 0.841 for HA means that 84.1% of the variance in HA is influenced by KH and PB, with values *β* = 0.169 and 0.791 respectively. Hence, KH and PB have a strong and significant impact on the halal awareness of MSE food producers.

According to Hair, *et al*., ^43^ *R* ^2^ values of 0.75, 0.50, or 0.25 for the endogenous construct, respectively, can be described as substantial, moderate, or weak. The *R* ^2^ value of an endogenous latent variable (i.e., halal awareness) described by the two predictive constructs in this study is 84.1% (see Figure 3), which is substantial. Hence, KH and PB are genuine predictors of halal awareness of food producers of MSE. The same goes for the *R* ^2^ value of the endogenous latent variable (i.e., Intention to register halal certification) is 82.5% which is substantial. However, the genuine predictor of MSEs’ Intention to register halal certification is only HA.

(v) *f* ^2^ effect size

In addition to evaluating the *R* ^2^ values of all endogenous constructs, the contribution of an exogenous latent variable to the *R* ^2^ value of an endogenous latent variable, known as the *f* ^2^ effect size, must be evaluated. In simpler words, the *f* ^2^ effect size measures the strength of the relationship between the latent variables. ^43^ Table 13 displays the *f* ^2^ values for all endogenous construct combinations (represented by columns) and corresponding exogenous (i.e., predictor) construct combinations (represented by the rows). As can be seen, HA has a large effect of 0.462 on IHC, while PB has a large effect of 2,035 on HA. Meanwhile, AHC (0.037) and PP (0.033)) have a medium effect on IHC, and KH (0.093) has a medium effect on HA.

(vi) Predictive relevance *Q* ^2^

Predictive relevance is another aspect that can be investigated for the inner model. The value of cross-validated redundancy ( *Q* ^2^) can be used to calculate the predictive value of relevance. If the *Q* ^2^ value is greater than zero it indicates that the model has predictive relevance accurate to certain constructs, otherwise the model lacks predictive relevance. ^43^

**Table 13.** The *f* ^2^ effect size of the final path model.

	HA	IHC
**AHC**		0.037
**HA**		0.462
**IHC**		
**KH**	0.093	
**PB**	2.035	
**PP**		0.033

The blindfolding procedure in SmartPLS can be used to calculate the *Q* ^2^ value. The output of the blindfolding procedure of the model discussed is shown in Table 14. In the table, SSO represents the sum of squared observations, SSE represents the sum of squared prediction errors, and the final column (i.e., 1 - SSE/SSO) represents the final value *Q* ^2^, which is interpreted to assess the model’s predictive relevance for each endogenous construct.

**Table 14.** The *Q* ^2^ value of the final path model.

	SSO	SSE	$Q^{2}=1-\frac{\mathrm{SSE}}{\mathrm{SSO}}$
**AHC**	548.000	548.000	
**HA**	411.000	137.423	0.666
**IHC**	411.000	114.042	0.723
**KH**	548.000	548.000	
**PB**	548.000	548.000	

As can be seen, the *Q* ^2^ values of two endogenous constructs (HA and IHC) are significantly higher than zero where the *Q* ^2^ value of IHC is greater than HA. These findings provide strong support for the model’s predictive relevance for endogenous latent variables. Hence, predictions for HA and IHC are accurate.

## Discussion

As can be seen in Table 5, the findings of the descriptive statistics indicate that most of the respondents are highly aware to have a halal certificate because they have a high level of knowledge of halal and generally agree that Halal Food Certification provides benefits. These are evidenced by a total mean KH of 4.621 and PB of 4.584 on a 5-point scale. These findings have confirmed the findings of Waluyo, ^27^ that religious knowledge and motivation to benefit have a significant impact on the awareness of food producers to certify their products. The finding that knowledge of halal has a significant impact on halal awareness is different from the finding of Giyanti and Indriastiningsih. ^16^

However, the descriptive statistics of perception of procedures (PP) revealed that obtaining a halal certificate is prohibitively expensive (the total mean PP of 3.159 on a 5 scale in Table 5). This has prompted MSE food producers to raise the price of their halal-certified products as a consequence of spending the cost of obtaining a halal certificate. However, they also worry that this price increase will lead to fewer sales. As a result, they think that obtaining a halal certificate is unnecessary. They assumed that their business would run smoothly even without the halal certificate. These findings align with the findings of Prabowo *et al.* ^13^

As shown in Figure 3, halal awareness and intention to register halal certification have a correlation coefficient of0.696. It means that the awareness of halal certification is a strong indicator of the intention to register halal certification. It aligns with Bachok *et al*., ^24^ Lee and Shin, ^25^ and Rezai *et al*. ^26^ The descriptive statistics also show that most of the respondents have a high intention to register halal certification with a total mean of 4.544 on a 5 scale ( Table 5).

This study finds that attitudes to produce halal products (AHC) and perception of halal certification procedures (PP) have a positive correlation but both do not significantly affect intentions to register halal certificates (IHC), as can be seen in Table 12. According to the Planned Behavior Theory, when there is support and a sense of ease that there are no barriers to behaviour, the intention to behave will increase. ^37^ This study shows that intention to register a halal certificate is not supported by the attitude because West Java MSE food producers perceive the procedures to obtain halal certification to be complex.

Another factor that hinders the desire to obtain a halal certificate is the lack of consumer pressure. Furthermore, MSE food producers are unaware of the risks of violating the halal product guarantee law if they do not have a halal certificate. This is alleged to lead to that attitude to produce halal foods having little bearing on their desire to register for halal certification.

These are the study’s main findings. According to our observation, the local community culture and mindset of micro and small-scale food entrepreneurs appear to be driving this lack of intention to obtain a halal certificate. Central Bureau of Statistics data, 61.63% of MSEs in West Java are owned by entrepreneurs with an elementary school education or less, and 42% have an annual income of 10 to 24 million rupiahs. ^18^ In general, West Java MSEfood producers are low- to middle-income communities with limited educational opportunities, so they rarely have broad perspectives or are willing to progress and develop. They are content to be able to sell every day without considering expanding their business.

Furthermore, by selling halal products, they have aided people who follow the Islamic faith to practice the Quran surah (chapter) 2:(Al Baqarah, ayah (verse) 168 ^32^:

*O mankind! Eat from whatever is on the earth - lawful and good and do not follow the footsteps of Shaitaan devil. Indeed, he is your clear enemy.*

The MSE food producers’ perception of complicated and costly certification procedures for their scale of operation also hampered their desire to register for halal certification. This finding supports Giyanti and Indriastiningsih, ^16^ and Abdul *et al*., ^19^ whose study found that MSEs’ food awareness/intention is hampered by a lack of socialization and the complexity of the procedure for handling halal certification.

According to the findings, the majority of MSE food producers have a negative perception of the government-mandated halal certification. The perception of complex procedures and relatively high costs are among the reasons. Furthermore, there is no consumer pressure regarding the halalness of the food to be purchased, and the limited MSE food producer education level creates the perception that their business can run smoothly without a halal certificate.

The MSE food producers are unaware that failing to have a halal certificate for their food products constitutes a violation of Law No. 33 2014 concerning halal product guarantees, which requires halal mandatory for food and beverages beginning October 17, 2024. These negative perceptions had to be fixed for UMK food producers to be ready to meet halal mandatory. Hence, the MSE food producers should be educated on the benefits and ease of obtaining a halal certificate. The Government of Indonesia is taking several educational steps, including socializing the benefits of halal certificates, assisting and providing financial support for MSE food producers to process halal certificates, and issuing rules regarding self-declaration.

The government launched a free halal certification program (called “Program Sehati”) through the Minister of Religion to arrange halal certificates for MSE food producers from March to December 2022 ^51^ and throughout 2023 by providing a self-declare scheme as regulated in PP No. 33 of 2021 concerning Implementation of the Halal Product Assurance Field. The government has provided a total of twenty-five thousand quotas self-declare scheme during 2022 ^51^ and one million quotas within 2023. ^52^ The government ensures that Micro, Small, and Medium Enterprises (MSMEs) can self-declare in order to obtain halal certification through this regulation. It means they only need to state that their product meets the standards of the Halal Product Assurance Organizing Agency (BPJPH) to be certified. ^53^

To assist with the halal certification process, the government collaborates with Islamic community organizations or Islamic religious institutions that have legal entities and/or universities. ^54^ Several community services conducted by universities show that socialization and assistance in the halal certification process through government-created programs can increase MSE food producers' intention to register their products. Oemar *et al*. ^17^ proved that socialization and training on halal awareness, halal assurance systems, and halal certification increase the understanding and awareness about halal-certified food so that all participants intend to obtain a Halal Certificate after completing the training. Ahmadiyah *et al.* ^55^ demonstrated that halal certification dissemination and assistance can raise MSEs' awareness of managing distribution permits and/or certification of their mainstay products. According to Pardiansyah *et al.*, ^56^ socialization and assistance with a free halal certification “Sehati Program” through a self-declare scheme make SMEs aware of the Sehati program's existence and understand its procedures and mechanisms.

In addition to the efforts already made, the already high MSE food producers' halal awareness should be reinforced by the availability of a readily accessible halal information center and innovative halal ecosystems. The halal information center is intended to serve food producers to share relevant information about halal food, benefits, procedures, halal assurance systems, and everything else related to halal certification. The halal ecosystem can be formed through a food halal supply chain system that involves suppliers, producers, and distributors.

## Conclusions

It can be concluded that micro and small-scale food producers in West Java Province, Indonesia has a good level of awareness about halal food even though they do not have a halal certificate. They pay attention to the halalness of the material used and of its processing. However, the perception of the procedures to obtain halal certificates which are relatively complicated and expensive for micro and small-scale businesses discourage the MSEs to register halal certification.

The hypothesis test shows that knowledge and perception of benefits have positive and significant correlations to halal awareness. In addition, halal awareness, attitude to produce halal foods, and perception of the procedure have a positive influence on the intention to register halal certification. However, attitude to produce halal foods and perception of procedure do not have a significant impact, while halal awareness has a significant effect on intention. This shows that halal awareness among MSE food producers can increase the intention to register their products to be halal certified. However, the reality shows that many products sold in the market do not have halal certificates. It indicates that the halal awareness of MSE food producers does not have an impact on real actions to register halal certification. They will act when they gain real benefit/profit. Hence, the government is attempting to increase the intention of MSE food producers to obtain halal certificates through socialization and assistance in obtaining halal certificates, free certification program called “Program Sehati”, and a self-declare scheme.

Finally, we recognize that this study has some limitations, including 1) The study focuses on micro and small food businesses. Perhaps in the future, research can be done on medium-scale food businesses that have a unique character due to the entrepreneur’s higher level of education. 2) The research was conducted in West Java, which has a distinct culture and mindset. In the future, perhaps research can be conducted in other Indonesian provinces with different characteristics. 3) The research was conducted for the food producers. Perhaps, study on other products will be possible in the future. 4) There are six variables in this study. Other estimated variables may be added in the future.

Further research may be undertaken to measure the level of halal awareness and intention to obtain the halal certificate for medium-scale entrepreneurs with other kinds of products in the other provinces and consider other related variables.

## Data availability

### Underlying data

Figshare: Dataset of Questionnaire Results from the respondents of Awareness and Intention to Register Halal Certification; https://doi.org/10.6084/m9.figshare.20488317. ^47^

This project includes the following underlying data:

- Questionnaire results from 137 West Java MSE food producers.

Data are available under the terms of the Creative Commons Attribution 4.0 International license (CC-BY 4.0).

Figshare: Dataset of West Java MSE food and culinary in 2021; https://doi.org/10.6084/m9.figshare.21618096. ^40^

This project includes the following underlying data:

• A copy of a list of West Java MSE food and culinary in 2021.

Data are available under the terms of the Creative Commons Attribution 4.0 International license (CC-BY 4.0).

### Extended data

Figshare: List of questions of descriptions of the questionnaire of Awareness and Intention to Register Halal Certification; https://doi.org/10.6084/m9.figshare.20488590. ^42^

This project includes the following extended data:

- A copy of the questionnaire

Data are available under the terms of the Creative Commons Attribution 4.0 International license (CC-BY 4.0).

Figshare: The Profile of Respondent of Awareness and Intention to Register Halal Certification; https://doi.org/10.6084/m9.figshare.20488650. ^57^

This project includes the following extended data:

- Profile of respondents

Data are available under the terms of the Creative Commons Attribution 4.0 International license (CC-BY 4.0).
