# Supplementary material for: Awareness and intention to register halal certification of micro and small-scale food enterprises
Source: F1000Res. 2023 Mar 29;11:170. Originally published 2022 Feb 11. [Version 3] doi: 10.12688/f1000research.75968.3 (PMC10526118; doi:10.12688/f1000research.75968.3)
Supplement: Peer review history

# Reviewer report: Reviewer response for version 3

**DOI:** 10.5256/f1000research.145658.r199459

**Published:** 2023-09-15

**Authors:** Salini Devi Rajendran

**Affiliations:** 1 School of Food Studies & Gastronomy Faculty of Social Sciences & Leisure Management, Taylor’s University, Kuala Lumpur, Malaysia

1. The authors should consider citing recent articles from well-known scholars who have studied the halal field to establish the credibility and relevance of their research.
2. The authors should elaborate more on the background of the halal industry in Indonesia. 
3. For the conclusion section, need more details in terms of implications. 

Is the work clearly and accurately presented and does it cite the current literature?

Partly

If applicable, is the statistical analysis and its interpretation appropriate?

Yes

Are all the source data underlying the results available to ensure full reproducibility?

Yes

Is the study design appropriate and is the work technically sound?

Yes

Are the conclusions drawn adequately supported by the results?

Partly

Are sufficient details of methods and analysis provided to allow replication by others?

Yes

Reviewer Expertise:

My research areas focus on food supply chain systems, halal product management, and sustainability. I am expertise in quantitative methodology and analysis including SPSS, PLS-SEM, and bibliometric analysis.

I confirm that I have read this submission and believe that I have an appropriate level of expertise to confirm that it is of an acceptable scientific standard.

# Reviewer report: Reviewer response for version 2

**DOI:** 10.5256/f1000research.137964.r153402

**Published:** 2022-12-21

**Authors:** Ai Chin Thoo

**Affiliations:** 1 Azman Hashim International Business School, Universiti Teknologi Malaysia, Skudai, Malaysia

Good job for the amendments.

 I think you use purposive sampling, not convenience sampling technique. Please check and amend.

Is the work clearly and accurately presented and does it cite the current literature?

Partly

If applicable, is the statistical analysis and its interpretation appropriate?

Partly

Are all the source data underlying the results available to ensure full reproducibility?

Partly

Is the study design appropriate and is the work technically sound?

Partly

Are the conclusions drawn adequately supported by the results?

Partly

Are sufficient details of methods and analysis provided to allow replication by others?

Partly

Reviewer Expertise:

marketing and supply chain management

I confirm that I have read this submission and believe that I have an appropriate level of expertise to confirm that it is of an acceptable scientific standard.

# Response

**Published:** 2023-03-15

**Authors:** Endang Prasetyaningsih

**Affiliations:** Universitas Islam Bandung, Indonesia

We appreciate your precious time in reviewing our paper and providing valuable comments.

# Reviewer report: Reviewer response for version 2

**DOI:** 10.5256/f1000research.137964.r153401

**Published:** 2022-11-08

**Authors:** Yukichika Kawata

**Affiliations:** 1 Faculty of Economics, Kindai University, Higashiosaka, Japan

Thank you for your modifications and detailed responses. Most of my concerns seem to be resolved appropriately. However, I still have a major concern about the process of gathering data. For example, the authors need to specify how readers can access " *the Central Bureau of Statistics of West Java*”, which provides the population information for this study. Some important factors, such as “ *predetermined criteria*”, are not explained, although more than half of the respondents are removed from the analyses. It is also unclear if " *the members of the West Java MSME association's WhatsApp groups*" coincide with " *680 entrepreneurs*". As indicated in S12, the authors should check non-response bias based on the comparison of the population and respondents, and not earlier and later respondents. Furthermore, the authors should discuss possible biases attributed to non-responses and whether the current conclusions hold. For example, the authors, in the discussion section, stated the following:

 “ *In general, West Java MSEfood producers are low- to middle-income communities with limited educational opportunities, so they rarely have broad perspectives or are willing to progress and develop.*”

 If those with limited educational opportunities are mainly removed when selecting 137 respondents, the authors can conclude that the above conclusion holds.

 Please see the additional comments below.

**M4)**

*The explanation of the basic information regarding food micro and small-scale enterprises (food MSEs) in West Java Province is inadequate. The authors state that: “According to our observations, the majority of micro and small-scale food entrepreneurs in West Java purchase raw materials and process them into finished products without storing and shipping."*

*Is the authors’ main target traditional restaurants such as “Kaki lima,” “Warung,” and “Rumah makan”? Please explain the types of food MSEs in greater detail. Which of the following are included under food MSEs in this study: producers, wholesalers, retailers, distributors, or restaurants? Also, please specify or give examples of what foods (e.g., carcasses at the middle, or dressed meats at almost the final stage of the food system) MSEs mainly treat.*

***Response:***

*We revised the statement to be:*

*“The study was carried out in West Java Province, Indonesia. The population is the MSE food producers listed in the Central Bureau of Statistics of West Java, such as producers of cassava chips, shredded catfish, “bagelen” cakes, chocolate “rangginang”, candied vegetables, market snacks, and so on.”*

**Comment on the 2nd round**

 The source of the Central Bureau of Statistics of West Java is not mentioned. Please provide information in the References section or footnotes to enable readers to access the original lists.

**S11)**

*Please also explain how many responses are considered: "376 people", "137 questionnaires", or "100 questionnaires"? If either 137 or 100 questionnaires are used, the results may be inaccurate if only earlier replies are considered for examination. This would hinder possible differences among respondents and may potentially cause biases.*

*I also did not understand the procedure followed while gathering questionnaires. The following two descriptions appear inconsistent:*

*“As a result, the data collected reached 100 respondents in just a matter of days. Due to the limitations of the software features used, the distribution of the questionnaire was halted. As a result, all collected data were subjected to validity and reliability tests."*

*“The questionnaires were distributed electronically to the West Java MSME community. The questionnaires were returned by 376 people. The returned questionnaires were then sorted using predetermined criteria, yielding 137 questionnaires that met the criteria. However, due to the limitations of the software features used, this study only processed 100 questionnaires at random."*

***Response:***

*We revised the explanation as follows:*

*“The questionnaires were distributed to the members of the West Java MSME association's WhatsApp groups. The questionnaires were returned by 376 respondents. The returned questionnaires were then sorted using predetermined criteria, yielding 137 respondents that met the criteria.”*

*“Finally, we decided to distribute the electronic questionnaire Google Forms in May 2020 to the West Java MSME association's WhatsApp groups. In just a few days, the data collected reached 137 respondents who met the predetermined criteria, so all collected data were subjected to validity and reliability tests.”*

**Comment on the 2nd round**

 WhatsApp group is newly mentioned in this revised manuscript. The relationship between 680 entrepreneurs (approximately 68% of the local association's 1000 members) and this WhatsApp group is unclear. Please add an explanation.

 A total of 137 respondents are selected from among 376 respondents based on the predetermined criteria. More than half of the respondents are removed in this process. Please explain the “ *predetermined criteria*" and justify the procedure of removing such a large number of respondents. Please also discuss biases that may occur in this procedure.

**S12)**

*Please add the corresponding population statistics to Table 7 and briefly discuss the possible biases, if any.*

*“Table 7 displays the percentage of respondents for each indicator. As shown in Table 7, 98% of respondents have Islam as their religion (Muslim), 71% are female, and 88% are 26 years old or older."*

***Response:***

*We tested the non-biased response, and the results are as follows:*

*“We examined the responses of early and late respondents to see if there was any possibility of non-response bias. According to Lindner et al.,44 respondents were classified into two groups:*

*early and late respondents. Late respondents were operationally and arbitrarily defined as the last half of respondents. Because there were 137 respondents in this study, 69 were identified as early respondents and 68 as late respondents. Hence, the independent samples t-test was then used to compare the two groups' responses to Likert scale questions. The findings show that there is no significant difference in key metrics responses between early and late respondents.”*

**Comment on the 2nd round**

 The earlier and later respondents may not be relevant when checking non-respondent bias. A total of 137 respondents may be compared with, for example, 376 respondents or 680 entrepreneurs. If population data are not available, authors can explain this accordingly.

**S18)**

*I do not understand the following paragraph. Did the MSE find it beneficial or unnecessary to obtain halal certificates?*

*“The findings also show that MSEs are aware of the benefits of the halal certificate. Based on our observation, we find that they do not require a halal certificate because they have satisfied with the sales/performance that has been achieved."*

***Response:***

*The explanation has been revised as follows:*

*“The findings also indicate that MSE food producers are aware of the benefits of the halal certificate. However, our observation revealed that they perceive obtaining a halal certificate as prohibitively expensive. It will cause the halal-certified product's selling price to rise. They are concerned that increasing selling prices will reduce sales. As a result, they perceive that obtaining a halal certificate is unnecessary. In any case, their business is running smoothly without the halal certificate. It aligns with the findings of Prabowo et al.13”*

**Comment on the 2nd round**

 The authors, in the Introduction, state that “ *[t]he Indonesian government has mandated that food producers have halal certificates to protect consumers.*” However, in the aforementioned response, the authors suggest that MSE producers can refuse to obtain halal certificates. It might be beneficial, for international readers, to explain why MSE producers can refuse to obtain halal certificates in defiance of the law.

Is the work clearly and accurately presented and does it cite the current literature?

Partly

If applicable, is the statistical analysis and its interpretation appropriate?

Partly

Are all the source data underlying the results available to ensure full reproducibility?

Partly

Is the study design appropriate and is the work technically sound?

Partly

Are the conclusions drawn adequately supported by the results?

Partly

Are sufficient details of methods and analysis provided to allow replication by others?

Yes

Reviewer Expertise:

applied economics

I confirm that I have read this submission and believe that I have an appropriate level of expertise to confirm that it is of an acceptable scientific standard, however I have significant reservations, as outlined above.

# Response

**Published:** 2023-03-15

**Authors:** Endang Prasetyaningsih

**Affiliations:** Universitas Islam Bandung, Indonesia

Thank you for your modifications and detailed responses. Most of my concerns seem to be resolved appropriately.

***Response:***

*We appreciate your valuable comments, which led to potential improvements in the current version. We carefully considered the comments and attempted to address each one. We hope that the manuscript will meet your high standards after careful revisions.*

 However, I still have a major concern about the process of gathering data. For example, the authors need to specify how readers can access "the Central Bureau of Statistics of West Java”, which provides the population information for this study.

***Response:***

*We have added data of the West Java MSE food and culinary 2021 obtained from the Central Bureau of Statistics of West Java, which has been uploaded to figshare and can be accessed online.*

 Some important factors, such as “predetermined criteria”, are not explained, although more than half of the respondents are removed from the analyses.

***Response:***

*We revised "predetermined criteria" to "determined criteria" to make it clear, hopefully. We add the following description of business scale categorization based on applicable regulations:*

*Categorization of business scale refers to the "Undang-Undang Republik Indonesia Nomor 20/2008” (Law of the Republic of Indonesia Number 20/2008).* ^39^ *According to that law, the criteria for micro-enterprises are as follows: *

1. *having a maximum net asset of Rp. 50,000,000.00 (fifty million rupiahs), excluding land and buildings for business premises; or *
2. *having a maximum annual sales turnover of IDR 300,000,000.00 (three hundred million rupiahs).*

*Whereas in article 6 paragraph 2, it is stated that the criteria for small businesses are as follows: *

1. *having a net asset of more than IDR 50,000,000.00 (fifty million rupiahs) up to a maximum of IDR 500,000,000.00 (five hundred million rupiahs) excluding land and buildings for business premises; or*
2. *having an annual sales turnover of more than IDR 300,000,000.00 (three hundred million rupiahs) up to IDR 2,500,000,000.00 (two billion five hundred million rupiahs).*

*MSE food producers chosen as respondents include those who meet the legal criteria. Furthermore, the purpose of this study is to determine the awareness level of MSE food producers regarding halal certification. Hence, the determined criteria for respondents were as follows:*

1. *a food producer of micro and small-scale with annual sales turnover of fewer than 2,500 million Rupiahs and net assets of fewer than 500 million Rupiahs; *
2. *have an ongoing business, and *
3. *no halal certificate.*

It is also unclear if "the members of the West Java MSME association's WhatsApp groups" coincide with "680 entrepreneurs".

***Response:***

*We explain this in the comment of S11.*

 As indicated in S12), the authors should check non-response bias based on the comparison of the population and respondents, and not earlier and later respondents. Furthermore, the authors should discuss possible biases attributed to non-responses and whether the current conclusions hold. For example, the authors, in the discussion section, stated the following:

 “In general, West Java MSEfood producers are low- to middle-income communities with limited educational opportunities, so they rarely have broad perspectives or are willing to progress and develop.”

 If those with limited educational opportunities are mainly removed when selecting 137 respondents, the authors can conclude that the above conclusion holds.

***Response:***

*We re-examined the non-response bias as described in response to S12.*

 Please see the additional comments below.

**M4)**

 The explanation of the basic information regarding food micro and small-scale enterprises (food MSEs) in West Java Province is inadequate. The authors state that: *“*According to our observations, the majority of micro and small-scale food entrepreneurs in West Java purchase raw materials and process them into finished products without storing and shipping."

 Is the authors’ main target traditional restaurants such as “Kaki lima,” “Warung,” and “Rumah makan”? Please explain the types of food MSEs in greater detail. Which of the following are included under food MSEs in this study: producers, wholesalers, retailers, distributors, or restaurants? Also, please specify or give examples of what foods (e.g., carcasses at the middle, or dressed meats at almost the final stage of the food system) MSEs mainly treat.

***Response:***

*We revised the statement to be:*

*“The study was carried out in West Java Province, Indonesia. The population is the MSE food producers listed in the Central Bureau of Statistics of West Java, such as producers of cassava chips, shredded catfish, “bagelen” cakes, chocolate “rangginang”, candied vegetables, market snacks, and so on.”*

**Comment on the 2nd round**

 The source of the Central Bureau of Statistics of West Java is not mentioned. Please provide information in the References section or footnotes to enable readers to access the original lists.

***Response:***

*A copy of the list of West Java MSEs registered in the Office of Cooperatives and Small Businesses in West Java Province obtained from the Central Bureau of Statistics of West Java can be found in Underlying Data ^2^.*

*Underlying Data:*

*Figshare: Dataset of West Java MSE food and culinary in 2021 ^2^*

**S11)**

 Please also explain how many responses are considered: "376 people", "137 questionnaires", or "100 questionnaires"? If either 137 or 100 questionnaires are used, the results may be inaccurate if only earlier replies are considered for examination. This would hinder possible differences among respondents and may potentially cause biases.

 I also did not understand the procedure followed while gathering questionnaires. The following two descriptions appear inconsistent:

 “As a result, the data collected reached 100 respondents in just a matter of days. Due to the limitations of the software features used, the distribution of the questionnaire was halted. As a result, all collected data were subjected to validity and reliability tests."

 “The questionnaires were distributed electronically to the West Java MSME community. The questionnaires were returned by 376 people. The returned questionnaires were then sorted using predetermined criteria, yielding 137 questionnaires that met the criteria. However, due to the limitations of the software features used, this study only processed 100 questionnaires at random."

***Response:***

*We revised the explanations as follows:*

*“The questionnaires were distributed to the members of the West Java MSME association's WhatsApp groups. The questionnaires were returned by 376 respondents. The returned questionnaires were then sorted using predetermined criteria, yielding 137 respondents that met the criteria.”*

*“Finally, we decided to distribute the electronic questionnaire Google Forms in May 2020 to the West Java MSME association's WhatsApp groups. In just a few days, the data collected reached 137 respondents who met the predetermined criteria, so all collected data were subjected to validity and reliability tests.”*

**Comment on the 2nd round**

 WhatsApp group is newly mentioned in this revised manuscript. The relationship between 680 entrepreneurs (approximately 68% of the local association's 1000 members) and this WhatsApp group is unclear. Please add an explanation.

***Response:***

*The West Java MSME association, which has over 1,000 members with 680 food and beverages entrepreneurs, has set up several WhatsApp groups to help them communicate with one another. Some groups exist due to the limited number of members who can join a single WhatsApp group. We collect data through these WhatsApp groups. A Google Form with a cover letter and a set of questionnaires were sent out electronically to the potential respondents who are members of the West Java MSME association's WhatsApp groups without separating food and non-food entrepreneurs.*

 A total of 137 respondents are selected from among 376 respondents based on the predetermined criteria. More than half of the respondents are removed in this process. Please explain the “predetermined criteria" and justify the procedure of removing such a large number of respondents. Please also discuss biases that may occur in this procedure.

***Response:***

*The questionnaires were distributed to the members of the West Java MSME association’s WhatsApp groups. The questionnaires were returned by 376 respondents, including food and non-food entrepreneurs with micro, small, and medium-scale businesses. All respondents who returned the questionnaires were then selected based on the determined criteria with the following stages:*

1. *divide them into two groups: those with medium-sized businesses and those with small or micro-scale businesses,*
2. *separate those with micro or small businesses from those with medium-scale businesses*
3. *choose the micro or small-scale businesses with food business product types.*
4. *select the micro or small-scale food producers who do not have halal certificates*

*Respondents who were chosen up to the fourth stage were referred to as “selected”, while the others as "non-selected". There were 137 selected respondents and 239 non-selected respondents as a result of the selection.*

**S12)**

 Please add the corresponding population statistics to Table 7 and briefly discuss the possible biases, if any.

 “Table 7 displays the percentage of respondents for each indicator. As shown in Table 7, 98% of respondents have Islam as their religion (Muslim), 71% are female, and 88% are 26 years old or older."

***Response:***

*We tested the non-biased response, and the results are as follows:*

*“We examined the responses of early and late respondents to see if there was any possibility of non- response bias. According to Lindner et al.,44 respondents were classified into two groups: early and late respondents. Late respondents were operationally and arbitrarily defined as the last half of respondents. Because there were 137 respondents in this study, 69 were identified as early respondents and 68 as late respondents. Hence, the independent samples t-test was then used to compare the two groups' responses to Likert scale questions. The findings show that there is no significant difference in key metrics responses between early and late respondents.”*

**Comment on the 2nd round**

 The earlier and later respondents may not be relevant when checking non-respondent bias. A total of 137 respondents may be compared with, for example, 376 respondents or 680 entrepreneurs. If population data are not available, authors can explain this accordingly.

***Response:***

*We examined the responses of the selected and non-selected respondents to see if there was any possibility of non-response bias. Lindner et al.,* ^44^ *proposed three methods for investigating non-response bias: (1) comparing early to late respondents, (2) using "days to respond" as a regression variable, and (3) comparing respondents to non-respondents. This study adopted the third proposed method of Lindner et al.,* ^44^ *by comparing selected and non-selected respondents. We examine 20% of both selected and non-selected respondents. That is, 27 respondents (20% of 137) were taken from the selected group, while 47 respondents (20% of 239) were taken from the non-selected group.*

*The independent samples t-test was used to compare the responses of the two groups due to the difference in the number of respondents examined. The findings show that there is no bias when none of the respondents in the two groups tested registered their products to obtain a halal certificate. When the non-selected respondents were a mix of those who had not yet registered and those who had already obtained halal certificates, two indicators were discovered to be biased in the perception of procedure variable (PP), namely PP2 and PP4 indicators, or in the perception of benefit variable (PB), namely PB4 indicator. This indicates that respondents who have obtained halal certificates have different perceptions than those who have not, particularly regarding perceptions of simplicity (PP2) and the length of time to obtain a halal certificate (PB4), as well as benefits such as increased competitiveness from having a halal certificate.*

**S18)**

 I do not understand the following paragraph. Did the MSE find it beneficial or unnecessary to obtain halal certificates?

 “The findings also show that MSEs are aware of the benefits of the halal certificate. Based on our observation, we find that they do not require a halal certificate because they have satisfied with the sales/performance that has been achieved."

**Response:**

 The explanation has been revised as follows:

 “The findings also indicate that MSE food producers are aware of the benefits of the halal certificate. However, our observation revealed that they perceive obtaining a halal certificate as prohibitively expensive. It will cause the halal-certified product's selling price to rise. They are concerned that increasing selling prices will reduce sales. As a result, they perceive that obtaining a halal certificate is unnecessary. In any case, their business is running smoothly without the halal certificate. It aligns with the findings of Prabowo et al.13”

**Comment on the 2nd round**

 The authors, in the Introduction, state that “[t]he Indonesian government has mandated that food producers have halal certificates to protect consumers.” However, in the aforementioned response, the authors suggest that MSE producers can refuse to obtain halal certificates. It might be beneficial, for international readers, to explain why MSE producers can refuse to obtain halal certificates in defiance of the law.

***Response:***

*According to the findings, the majority of MSE food producers have a negative perception of the government-mandated halal certification. The perception of complex procedures and relatively high costs are among the reasons. Furthermore, there is no consumer pressure regarding the halalness of the food to be purchased, and the limited MSE food producer education level creates the perception that their business can run smoothly without a halal certificate.*

*The MSE food producers are unaware that failing to have a halal certificate for their food products constitutes a violation of Law No. 33 2014 concerning halal product guarantees, which requires halal certification for food and beverages beginning October 17, 2024. These negative perceptions have to be fixed for UMK food producers to be ready to meet halal certification. Hence, the MSE food producers should be educated on the benefits and ease of obtaining a halal certificate. The Government of Indonesia is taking several educational steps, including socializing the benefits of halal certificates, assisting and providing financial support for MSE food producers to process halal certificates, and issuing rules regarding self-declaration.*

*The government launched a free halal certification program (called "Program Sehati") through the Minister of Religion to arrange halal certificates for MSE food producers from March to December 2022* *^3^ and throughout 2023 by providing a self-declare scheme as regulated in PP No. 33 of 2021 concerning Implementation of the Halal Product Assurance Field. The government has provided a total of twenty-five thousand quotas through the self-declare scheme during 2022 ^3^* *and one million quotas within 2023.* *^4^* *The government ensures that Micro, Small, and Medium Enterprises (MSMEs) can self-declare in order to obtain halal certification through this regulation. It means they only need to state that their product meets the standards of the Halal Product Assurance Organizing Agency (BPJPH) to be certified.* *^5^*

*To assist with the halal certification process, the government collaborates with Islamic community organizations or Islamic religious institutions that have legal entities and/or universities.* *^6^* *Several community services conducted by universities show that socialization and assistance in the halal certification process through government-created programs can increase MSE food producers' intention to register their products. Oemar et al.* ^17^ *proved that socialization and training on halal awareness, halal assurance systems, and halal certification increase the understanding and awareness about halal-certified food so that all participants intend to obtain a Halal Certificate after completing the training. Ahmadiyah et al.* *^7^* *demonstrated that halal certification dissemination and assistance can raise MSEs' awareness of managing distribution permits and/or certification of their mainstay products. According to Pardiansyah et al.* *^8^* *,* *socialization and assistance with a free halal certification "Sehati Program" through a self-declare scheme make SMEs aware of the Sehati Program's existence and understand its procedures and mechanisms.*

*In addition to the efforts already made, the already high MSE food producers' halal awareness should be reinforced by the availability of a readily accessible halal information center and innovative halal ecosystems. The halal information center is intended to serve food producers to share relevant information about halal food, benefits, procedures, halal assurance systems, and everything else related to halal certification. The halal ecosystem can be formed through a food halal supply chain system that involves suppliers, producers, and distributors.*

# Reviewer report: Reviewer response for version 1

**DOI:** 10.5256/f1000research.79916.r127721

**Published:** 2022-03-31

**Authors:** Ai Chin Thoo

**Affiliations:** 1 Azman Hashim International Business School, Universiti Teknologi Malaysia, Skudai, Malaysia

The practical gaps are not aligned with the research objectives. What is the relationship between the global market and the intention of local MSEs for the halal certificates? Need to include practical and theoretical gaps in the Introduction part.

 The literature review is not coherently discussed. Lack of justifications for the hypotheses development. 

 The population, sample, sample size, sampling technique, and data collection procedure need to be highlighted and discussed in detail. 

 Please justify why 100 samples were used for data analysis. ' *The questionnaires were distributed electronically to the West Java MSME community. The questionnaires were returned by 376 people. The returned questionnaires were then sorted using predetermined criteria, yielding 137 questionnaires that met the criteria. However, due to the limitations of the software features used, this study only processed 100 questionnaires at random*.'

Is the work clearly and accurately presented and does it cite the current literature?

Partly

If applicable, is the statistical analysis and its interpretation appropriate?

Partly

Are all the source data underlying the results available to ensure full reproducibility?

Partly

Is the study design appropriate and is the work technically sound?

Partly

Are the conclusions drawn adequately supported by the results?

Partly

Are sufficient details of methods and analysis provided to allow replication by others?

Partly

Reviewer Expertise:

marketing and supply chain management

I confirm that I have read this submission and believe that I have an appropriate level of expertise to confirm that it is of an acceptable scientific standard, however I have significant reservations, as outlined above.

# Response

**Published:** 2022-08-29

**Authors:** Endang Prasetyaningsih

**Affiliations:** Universitas Islam Bandung, Indonesia

Thank you for your insightful comments.  Each comment is addressed in a point-by-point format.

 1. The practical gaps are not aligned with the research objectives. What is the relationship between the global market and the intention of local MSEs for the halal certificates? Need to include practical and theoretical gaps in the Introduction part.

**Response:**

 We have rewritten the introduction section.

 The purpose of this study is to assess the level of halal awareness and the intention of food producers in West Java Province Indonesia to register for halal certification.

 Hence, we rewrite the introduction section to describe the gap between the necessity of having a halal certificate and the negative perception of halal certification among food entrepreneurs in several Indonesian cities.

 2. The literature review is not coherently discussed. Lack of justifications for the hypotheses development. 

**Response:**

 We have rewritten the literature review section

 3. The population, sample, sample size, sampling technique, and data collection procedure need to be highlighted and discussed in detail. 

**Response:**

 We have added a new sub-section called "Population and Sample." and the data collection’s description.  

*“* *Population and sample*

*The study was carried out in West Java Province, Indonesia.* *The population is the MSE food producers listed in the Central Bureau of Statistics of West Java, such as producers of cassava chips, shredded catfish, “bagelen” cakes, chocolate “rangginang”, candied vegetables, market snacks, and so on* *.* *Categorization of business scale refers to the "Undang-Undang Republik Indonesia Nomor 20/2008” (Law of the Republic of Indonesia Number 20/2008).* *^39^* *Table 2 shows the c* *ategorization of the business scale.* *The population chosen consisted of those who met the criteria 1) a food producer of micro and small-scale with annual sales turnover of fewer than 2,500 Rupiahs and net assets of fewer than 500 million Rupiahs; 2) have an ongoing business, and 3) no halal certificate.*”

*The convenience sampling technique was used in this study. The total food producer of MSE listed in the Central Bureau of Statistics of West Java was estimated to be around 2300 people. We worked with "Sahabat UMKM Jawa Barat" (West Java MSME association-English), a local association of micro, small, and medium-scale entrepreneurs who are engaged in a variety of fields such as culinary, fashion, crafts, and other businesses or industries. The local association has over 1000 members, with 68 percent of them being food and beverage entrepreneurs, or approximately 680 entrepreneurs. This study's sample consists of food producers who meet the predetermined criteria among the 680 entrepreneurs.”*

*“Data collection*

*A questionnaire is chosen as the research instrument. The questionnaires were re-translated from English to Indonesian, except for those references that were already in Indonesian. Each variable is made up of measurement items which are scored on a Likert scale of 1 (strongly disagree) to 5 (strongly agree).*

*A Google Form with a cover letter and a set of questionnaires were sent out to the potential respondents who are members of the West Java MSME association WhatsApp groups. This method was chosen because of the COVID-19 pandemic outbreak. In addition, the designed questionnaires could be collected without conducting direct visits to the respondents. The respondents could not participate in the survey unless they gave their written consent. Data were collected from March to May 2020. A copy of the distributed questionnaires can be found in Extended Data.* *^40^* *The questionnaire was pretested with a small sample of members of the West Java MSME association before being distributed to the actual respondents. Based on pretest feedback, the wording of some items was refined and modified to guarantee that the validity and reliability of each variable meet the required standard. The question items were scored on a Likert scale of 1 (strongly disagree) to 5 (strongly agree). The follow-up of this plan is described later in the data preparation section.*

*The convenience sampling method is used in the following manner.*

1. *Distribute questionnaires, *
2. *Wait for responses to the distributed questionnaire,*
3. *Collect data until the sample count is adequate.*
4. *Screen participants based on the criteria specified.”*

4. Please justify why 100 samples were used for data analysis. ' *The questionnaires were distributed electronically to the West Java MSME community. The questionnaires were returned by 376 people. The returned questionnaires were then sorted using predetermined criteria, yielding 137 questionnaires that met the criteria. However, due to the limitations of the software features used, this study only processed 100 questionnaires at random*.'

**Response:**

 In the revised manuscript, we recalculated by increasing the number of respondents from 100 to 137 based on the reviewers' recommendations.

# Reviewer report: Reviewer response for version 1

**DOI:** 10.5256/f1000research.79916.r123266

**Published:** 2022-02-18

**Authors:** Yukichika Kawata

**Affiliations:** 1 Faculty of Economics, Kindai University, Higashiosaka, Japan

I found that the topics of this study reflect the current Indonesian status of halal certification and would attract the interest of both scholars and the general public. However, some significant issues remain unaddressed.

**Major comments:**

**M1)** The procedure for constructing the sample is not appropriate. The authors state that:

*“The questionnaires were returned by 376 people. The returned questionnaires were then sorted using predetermined criteria, yielding 137 questionnaires that met the criteria. However, due to the limitations of the software features used, this study only processed 100 questionnaires at random.”*

 It is not acceptable to select 100 out of 137 questionnaires, even if they are randomly selected. The limitations of the software may not be reasonable ground for such an exclusion because other open-source and academically-sound software such as “R" is available, which provides packages to conduct PLS-SEM or other SEMs.

**M2)** The explanations on how the final model is constructed are insufficient. For example, the authors do not report any indices of goodness-of-fit such as AIC, GFI, AGFI, and CFI.

**M3)** What is the purpose of this study? The main targets of this study are micro and small-scale food enterprises, and the authors state that:

*“Hence, this study aims at measuring the level of halal awareness and the intention of food MSE entrepreneurs in West Java Province, Indonesia to register halal certification.”*

 I do not understand why the authors repeatedly explain the situation of exports to other countries and the status of importing countries such as the Philippines. Such descriptions seem to be redundant. For example,

*“The globalization of the halal industry provides an opportunity for local micro and small-scale enterprises (MSEs) to sell halal products. Local MSEs, on the other hand, are hesitant to compete in the global market because they lack halal certificates, which are one of the most important requirements for entering the global market. Halal certifications are also regarded as a quality-control standard among Muslim consumers. Many non-Muslims have no qualms about eating halal food, but they may react negatively if they eat halal food accidentally and feel cheated.3 As a result, MSEs that want to enter the global market must have a halal certificate.”*

*“Non-halal restaurant owners in Manila, Philippines are generally aware of the 12 halal certification standards (raw materials, tools and equipment, facilities, buildings, exterior areas, location, halal documentation, staff characteristics, staff policies, pest controls, management responsibilities, and waste management), and the majority are ‘Willing’ to be halal certified.”*

*“Halal food certificates are required to increase self-confidence, customer trust, and customer satisfaction, although SMEs in Hat Yai, Thailand are dissatisfied with the poor dissemination of halal hub information.”*

 If the authors intend to examine the possibility of food exports by MSEs in West Java Province, the discussion section would require to be revised to meet such objectives.

**M4)** The explanation of the basic information regarding food micro and small-scale enterprises (food MSEs) in West Java Province is inadequate. The authors state that:

*“According to our observations, the majority of micro and small-scale food entrepreneurs in West Java purchase raw materials and process them into finished products without storing and shipping."*

 Is the authors’ main target traditional restaurants such as “Kaki lima,” “Warung,” and “Rumah makan”? Please explain the types of food MSEs in greater detail. Which of the following are included under food MSEs in this study: producers, wholesalers, retailers, distributors, or restaurants? Also, please specify or give examples of what foods (e.g., carcasses at the middle, or dressed meats at almost the final stage of the food system) MSEs mainly treat.

**Minor comments:**

**S1)** The authors state the following:

*“With the issuance of “Undang-Undang Republik Indonesia No 33 Tahun 2014” (Law of Republic Indonesia No 33/2014 - English)10 regarding halal product guarantees, the halal certification has become a requirement for producers in Indonesia.”*

 They also state,

*“Although Islam is the majority religion in Indonesia, most micro, small, and medium enterprises (MSMEs) do not register their products for halal certification. Only 10% of MSMEs have halal certificates, according to the Association of Food and Beverage Entrepreneurs (GAPMMI) in June 2019.”*

 It appears that these two explanations are contradictory. Kindly elucidate further.

**S2)** I do not agree with the following description,

*“These indicate the food MSEs' lack of understanding and awareness of the importance of halal products.”*

 It is untrue that food MSEs lack understanding and awareness of the importance of halal products. However, they lack the necessity to obtain halal certificates. Most target local markets and may not intend to export their products to Muslims in other countries. If so, they have no incentive to acquire a halal certification for trade within Indonesia or export outside Indonesia.

**S3)** Is the following extract from the paper related to “ *halal awareness*”? Since CSR is a broader concept, it might be beneficial for readers if you add an explanation of CSR in Islamic or Indonesian contexts.

*“Meanwhile, the procedure for obtaining halal certificates is relatively complex, thereby reducing the intention of SMEs to register halal certification. Meanwhile, Lee and Shin37 found that consumers' awareness of Corporate Social Responsibility (CSR) activities and purchase intentions are positively related."*

**S4)** The authors write “ *attitude to produce halal product (AHC)*” in the body text.

*“Referring to Giyanti and Indriastiningsih,14 Waluyo,19 and Lee and Shin,37 and the Planned Behavior Theory,32 this study identifies that the variables are knowledge of halal (KH), perception of benefits (PB), perception of procedures (PP), halal awareness (HA), attitude to produce halal product (AHC) and intention to register a halal certificate."*

 However, they write “ *Attitude to register halal product*” in Figure 1. Are they both correct?

**S5)** Please add a brief explanation on "green foods" and possibly also on the relationship between the halal concept and green foods.

*“Consumer awareness of the green concept is a strong predictor of their intention to consume green foods, ^17^ while customer awareness towards the halal logo fosters purchase intention."*

**S6)** Please explain the following in more detail:

*“Initially, 680 micro and small-scale food and beverage entrepreneurs in the West Java MSME community were targeted."*

 It is not clear if 680 is the total number of micro-and small-scale food and beverage entrepreneurs in West Java or if it is the number of entrepreneurs selected by the authors. Please consider adding the total number of micro, small, and medium food and beverage entrepreneurs in West Java in Table 1 and the number of micro and small entrepreneurs that were targets of this study.

**S7)** Please specify if those involved in the pilot survey are from West Java or other provinces.

*“The questionnaire was pretested with a small sample of food MSE entrepreneurs before being distributed to the actual respondents."*

**S8)** Please consider using "Partial Least Squares Structural Equation Modeling (PLS-SEM)” instead of “ *Structural Equation Model-Partial Least Square (SEM-PLS).*”

**S9)** The following literature is not cited in the body text:

*“40. Menteri Hukum dan HAM (Minister of Law and Human Rights): Undang-Undang Republik Indonesia Nomor 20 Tahun 2008 (Law of Republic Indonesia Number 20/2008).2008; 1–11."*

**S10)** Please consider quoting references for this procedure:

*“We analyzed the chi-square of early and late respondents' responses to see if there was any potential for non-response bias (the first and last 20 percent of responses received)."*

**S11)** Please also explain how many responses are considered: "376 people", "137 questionnaires", or "100 questionnaires"? If either 137 or 100 questionnaires are used, the results may be inaccurate if only earlier replies are considered for examination. This would hinder possible differences among respondents and may potentially cause biases.

 I also did not understand the procedure followed while gathering questionnaires. The following two descriptions appear inconsistent:

*“As a result, the data collected reached 100 respondents in just a matter of days. Due to the limitations of the software features used, the distribution of the questionnaire was halted. As a result, all collected data was subjected to validity and reliability tests."*

*“The questionnaires were distributed electronically to the West Java MSME community. The questionnaires were returned by 376 people. The returned questionnaires were then sorted using predetermined criteria, yielding 137 questionnaires that met the criteria. However, due to the limitations of the software features used, this study only processed 100 questionnaires at random."*

 As pointed out in the major comment, I do not agree with the use of 100 samples.

**S12)** Please add the corresponding population statistics to Table 7 and briefly discuss the possible biases, if any.

*“Table 7 displays the percentage of respondents for each indicator. As shown in Table 7, 98% of respondents have Islam as their religion (Muslim), 71% are female, and 88% are 26 years old or older."*

**S13)** Why do the authors not remove Christians from the selected 100 respondents? (Table 7 shows one Catholic and one Protestant.)

**S14)** Please consider aligning digits in Table 8, e.g., "3.2225" maybe "3.223", etc.

**S15)** Please specify the percentage selected by the authors as statistically significant (e.g., 5%) in Table 9.

**S16)** Please use a period instead of a comma as a decimal point. For example, the authors used both in the following paragraph (82,6% and 82.6%, respectively).

*“Besides, the R2 value of 0.826 for HA means that 82,6% of the variance in HA is influenced by KH and PB, with values β = 0,292 and 0,683 respectively. Hence, KH and PB have a strong and significant impact on the awareness of MSE entrepreneurs about the importance of halal products. According to Hair, et al.41 R2 values of 0.75, 0.50, or 0.25 for the endogenous construct, respectively, can be described as substantial, moderate, or weak. The R2 value of an endogenous latent variable (i.e., halal awareness) described by the two predictive constructs in this study is 82.6% (see Figure 2), which is substantial."*

**S17)** The intended meaning of the following sentence is ambiguous.

*“The finding that knowledge of halal has a significant impact on knowledge of halal is different from the finding of Giyanti and Indriastiningsih ^14^"*

 The phrase “ *knowledge of halal has a significant impact on knowledge of halal*” is not clear. Please consider rewording it.

 Please also add a period at the end of this sentence.

**S18)** I do not understand the following paragraph. Did the MSE find it beneficial or unnecessary to obtain halal certificates?

*“The findings also show that MSEs are aware of the benefits of the halal certificate. Based on our observation, we find that they do not require a halal certificate because they have satisfied with the sales/performance that has been achieved."*

 Similarly, I also do not understand why the authors conclude “ *[h]alal certificates will therefore increase sales and revenue*” in the following context? Please consider a more logical construction of the sentence.

*“They are unaware that halal certificates will increase consumer trust and help food businesses compete more effectively. Halal certificates will therefore increase sales and revenue."*

**S19)** Please explain the relationship between respondents of this study and “ *street vendors around Universitas Islam Bandung (Unisba)*” that is mentioned in the “Discussion” section.

*“Socialization and training on halal awareness, halal guarantee system, and halal certification for street vendors around Universitas Islam Bandung (Unisba) which is located at Bandung, the Capital of West Java Province Indonesia have been conducted by Oemar et al.13 The trainees gain a better understanding and awareness of halal food as a result of the training. Consequently, all trainees intend to obtain a Halal Certificate."*

**S 20)** Please recheck the references carefully. For example, you can quote my work (reference 9), but the publication year, pages, etc., are incorrect.

Is the work clearly and accurately presented and does it cite the current literature?

Partly

If applicable, is the statistical analysis and its interpretation appropriate?

Partly

Are all the source data underlying the results available to ensure full reproducibility?

Partly

Is the study design appropriate and is the work technically sound?

Partly

Are the conclusions drawn adequately supported by the results?

Partly

Are sufficient details of methods and analysis provided to allow replication by others?

Yes

Reviewer Expertise:

applied economics

I confirm that I have read this submission and believe that I have an appropriate level of expertise to confirm that it is of an acceptable scientific standard, however I have significant reservations, as outlined above.

# Response

**Published:** 2022-08-29

**Authors:** Endang Prasetyaningsih

**Affiliations:** Universitas Islam Bandung, Indonesia

Thank you for your insightful comments. Each comment is addressed in a point-by-point format.

**Major comments:**

**M1)** The procedure for constructing the sample is not appropriate. The authors state that:

*“The questionnaires were returned by 376 people. The returned questionnaires were then sorted using predetermined criteria, yielding 137 questionnaires that met the criteria. However, due to the limitations of the software features used, this study only processed 100 questionnaires at random.”*

 It is not acceptable to select 100 out of 137 questionnaires, even if they are randomly selected. The limitations of the software may not be reasonable ground for such an exclusion because other open-source and academically-sound software such as “R" is available, which provides packages to conduct PLS-SEM or other SEMs.

 Response:

 We recalculated by increasing the number of respondents from 100 to 137 in the revised manuscript.

**M2)** The explanations on how the final model is constructed are insufficient. For example, the authors do not report any indices of goodness-of-fit such as AIC, GFI, AGFI, and CFI.

 Response:

 PLS-SEM uses SRMS, and RMS _theta_ to test the model fit. We state that

*“* *The PLS-SEM model fit is evaluated using standardized root mean square residual (SRMR), root mean square residual covariance (RMS _theta_), or the exact fit test to determine how well it predicts endogenous variables/constructs.* *^41^* *”*

 We choose SRMR where the result is stated as follows

*“* *This study uses the standardized root means square (SMSR) to assess model fit. The SMSR is 0.067, less than 0.08, indicating that the model meets the model fit criteria. Furthermore, the data can be used to estimate the model.”*

**M3)** What is the purpose of this study? The main targets of this study are micro and small-scale food enterprises, and the authors state that:

*“Hence, this study aims at measuring the level of halal awareness and the intention of food MSE entrepreneurs in West Java Province, Indonesia to register halal certification.”*

 I do not understand why the authors repeatedly explain the situation of exports to other countries and the status of importing countries such as the Philippines. Such descriptions seem to be redundant. For example,

*“The globalization of the halal industry provides an opportunity for local micro and small-scale enterprises (MSEs) to sell halal products. Local MSEs, on the other hand, are hesitant to compete in the global market because they lack halal certificates, which are one of the most important requirements for entering the global market. Halal certifications are also regarded as a quality-control standard among Muslim consumers. Many non-Muslims have no qualms about eating halal food, but they may react negatively if they eat halal food accidentally and feel cheated.3 As a result, MSEs that want to enter the global market must have a halal certificate.”*

*“Non-halal restaurant owners in Manila, Philippines are generally aware of the 12 halal certification standards (raw materials, tools and equipment, facilities, buildings, exterior areas, location, halal documentation, staff characteristics, staff policies, pest controls, management responsibilities, and waste management), and the majority are ‘Willing’ to be halal certified.”*

*“Halal food certificates are required to increase self-confidence, customer trust, and customer satisfaction, although SMEs in Hat Yai, Thailand are dissatisfied with the poor dissemination of halal hub information.”*

 If the authors intend to examine the possibility of food exports by MSEs in West Java Province, the discussion section would require to be revised to meet such objectives.

 Response:

 The purpose of this study is not to investigate the possibility of food exports by MSEs in West Java Province, but to assess the level of halal awareness and the intention of food producers in West Java Province, Indonesia, to register for halal certification.

 Therefore, we rewrite the introduction section to describe the gap between the necessity of having a halal certificate and the negative perception of halal certification among food entrepreneurs in several Indonesian cities.

**M4)** The explanation of the basic information regarding food micro and small-scale enterprises (food MSEs) in West Java Province is inadequate. The authors state that:

*“According to our observations, the majority of micro and small-scale food entrepreneurs in West Java purchase raw materials and process them into finished products without storing and shipping."*

 Is the authors’ main target traditional restaurants such as “Kaki lima,” “Warung,” and “Rumah makan”? Please explain the types of food MSEs in greater detail. Which of the following are included under food MSEs in this study: producers, wholesalers, retailers, distributors, or restaurants? Also, please specify or give examples of what foods (e.g., carcasses at the middle, or dressed meats at almost the final stage of the food system) MSEs mainly treat.

 Response:

 We revised the statement to be:

*“* *The study was carried out in West Java Province, Indonesia.* *The population is the MSE food producers listed in the Central Bureau of Statistics of West Java, such as producers of cassava chips, shredded catfish, “bagelen” cakes, chocolate “rangginang”, candied vegetables, market snacks, and so on* *.”*

**Minor comments:**

**S1)** The authors state the following:

*“With the issuance of “Undang-Undang Republik Indonesia No 33 Tahun 2014” (Law of Republic Indonesia No 33/2014 - English)10 regarding halal product guarantees, the halal certification has become a requirement for producers in Indonesia.”*

 They also state,

*“Although Islam is the majority religion in Indonesia, most micro, small, and medium enterprises (MSMEs) do not register their products for halal certification. Only 10% of MSMEs have halal certificates, according to the Association of Food and Beverage Entrepreneurs (GAPMMI) in June 2019.”*

 It appears that these two explanations are contradictory. Kindly elucidate further.

 Response:

 We rewrite the introduction section and describe the contradiction.

*“* *Indonesia is a country where Islam is the majority religion. The Indonesian government has mandated that food producers have halal certificates to protect consumers, particularly Muslim consumers when purchasing food, by issuing Undang-Undang Republik Indonesia No 33 Tahun 2014" (Law of Republic Indonesia No 33/2014 - English) ^10^ regarding halal product guarantees. However, statistics show that the majority of food micro, small, and medium-scale enterprises (food MSMEs) do not register their products for halal certification. According to the Association of Food and Beverage Entrepreneurs (GAPMMI) in June 2019, only 10% of MSMEs have halal certificates.* *^10^* *This contradiction indicates that the intention of SMEs to sell halal-certified products remains low. They may be unaware of the benefits of halal certification or have a negative perception of halal certificates. Several studies on the perception of halal certificates have been conducted in various cities in Indonesia.”*

**S2)** I do not agree with the following description, *“These indicate the food MSEs' lack of understanding and awareness of the importance of halal products.”*

 It is untrue that food MSEs lack understanding and awareness of the importance of halal products. However, they lack the necessity to obtain halal certificates. Most target local markets and may not intend to export their products to Muslims in other countries. If so, they have no incentive to acquire a halal certification for trade within Indonesia or export outside Indonesia.

 Response:

 Focus on the purpose of our research; we do not discuss product export; rather, we measure awareness and intention to register for a halal certificate. Several studies on food entrepreneurs' perceptions of halal certification in several Indonesian cities are as follows.

*“* *The majority of MSME entrepreneurs in some cities of East Java Province, Indonesia, such as Bangkalan, Pamekasan, and Pasuruan, understand the significance of halal certificates. However, they consider halal certification to be unimportant.* *^12^* *In East Kalimantan Province, Indonesia the MSME entrepreneurs even underestimate the halal certificate due to believing that their business is running smoothly despite the lack of a halal certificate.* *^13^* *Meanwhile, entrepreneurs in other Indonesian cities such as the Greater Jakarta Area,* *^14^* *Malang City East Java Province,* *^15^* *and Surakarta City Central Java Province* *^16^* *did not register halal certification because they perceive the process as difficult and costly. These cases indicate MSME entrepreneurs’ lack of necessity for obtaining halal certificates.* *“*

**S3)** Is the following extract from the paper related to “ *halal awareness*”? Since CSR is a broader concept, it might be beneficial for readers if you add an explanation of CSR in Islamic or Indonesian contexts.

*“Meanwhile, the procedure for obtaining halal certificates is relatively complex, thereby reducing the intention of SMEs to register halal certification. Meanwhile, Lee and Shin37 found that consumers' awareness of Corporate Social Responsibility (CSR) activities and purchase intentions are positively related."*

 Response:

 We concentrate on the findings of the Lee and Shin ^24^ study, specifically the relationship between awareness and intention, rather than the case study. Hence, we updated the explanation as follows:

*“* *According to Liba et al.,* *^9^* *Elias et al.,* *^20^* *and Masithoh et al.,* *^21^* *awareness of halal is positively correlated with the intention to obtain halal certification. Meanwhile, Dinev and Hu,* *^22–25^* *Bachok et al.,* *^23^* *Lee and Shin,* *^24^* *Rezai et al.,* *^25^* *state that customer awareness is a strong predictor of a customer's intent to buy or select a product. These indicate that awareness influences intention.”*

**S4)** The authors write “ *attitude to produce halal product (AHC)*” in the body text.

*“Referring to Giyanti and Indriastiningsih,14 Waluyo,19 and Lee and Shin,37 and the Planned Behavior Theory,32 this study identifies that the variables are knowledge of halal (KH), perception of benefits (PB), perception of procedures (PP), halal awareness (HA), attitude to produce halal product (AHC) and intention to register a halal certificate."*

 However, they write “ *Attitude to register halal product*” in Figure 1. Are they both correct?

 Response:

 We revised the explanation and Figure 1.

**S5)** Please add a brief explanation on "green foods" and possibly also on the relationship between the halal concept and green foods.

*“Consumer awareness of the green concept is a strong predictor of their intention to consume green foods, ^17^ while customer awareness towards the halal logo fosters purchase intention."*

 Response:

 We are interested in the study's findings, specifically the relationship between awareness and intention, rather than the relationship between the halal concept and green foods. As a result, we revised the explanation as follows:

*“* *Consumer awareness is a strong predictor of their intention to consume/purchase foods.* *^23,25^* *”*

**S6)** Please explain the following in more detail:

*“Initially, 680 micro and small-scale food and beverage entrepreneurs in the West Java MSME community were targeted."*

 It is not clear if 680 is the total number of micro-and small-scale food and beverage entrepreneurs in West Java or if it is the number of entrepreneurs selected by the authors. Please consider adding the total number of micro, small, and medium food and beverage entrepreneurs in West Java in Table 1 and the number of micro and small entrepreneurs that were targets of this study.

 Response:

 We revise the explanation as follows:

*“The convenience sampling technique was used in this study. The total food producer of MSE listed in the Central Bureau of Statistics of West Java was estimated to be around 2300 people. We worked with "Sahabat UMKM Jawa Barat" (West Java MSME association-English), a local association of micro, small, and medium-scale entrepreneurs who are engaged in a variety of fields such as culinary, fashion, crafts, and other businesses or industries. The local association has over 1000 members, with 68 percent of them being food and beverage entrepreneurs, or approximately 680 entrepreneurs. This study's sample consists of food producers who meet the predetermined criteria among the 680 entrepreneurs.“*

**S7)** Please specify if those involved in the pilot survey are from West Java or other provinces.

*“The questionnaire was pretested with a small sample of food MSE entrepreneurs before being distributed to the actual respondents."*

 Response:

 We included the pilot survey participant in the following statement:

*“* *The questionnaire was pretested with a small sample of members of the West Java MSME association before being distributed to the actual respondents.”*

**S8)** Please consider using "Partial Least Squares Structural Equation Modeling (PLS-SEM)” instead of “ *Structural Equation Model-Partial Least Square (SEM-PLS).*”

 Response:

 As suggested, we have revised SEM-PLS to PLS-SEM.

**S9)** The following literature is not cited in the body text:

*“40. Menteri Hukum dan HAM (Minister of Law and Human Rights): Undang-Undang Republik Indonesia Nomor 20 Tahun 2008 (Law of Republic Indonesia Number 20/2008).2008; 1–11."*

 Response:

 We have quoted "Undang-Undang Republik Indonesia Nomor 20/2008” (Law of the Republic of Indonesia Number 20/2008). ^39^ in body text and Table 2 as follows:

*“* *Categorization of business scale refers to the "Undang-Undang Republik Indonesia Nomor 20/2008” (Law of the Republic of Indonesia Number 20/2008).* *^39^*”

*“Table 2. Category of micro, small and medium business in Indonesia according to Law of the Republic of Indonesia No. 20/2008.* *^39^* *”*

**S10)** Please consider quoting references for this procedure:

*“We analyzed the chi-square of early and late respondents' responses to see if there was any potential for non-response bias (the first and last 20 percent of responses received)."*

 Response:

 We discovered an appropriate reference in our research and changed the statement to:

*“* *We examined the responses of early and late respondents to see if there was any possibility of non-response bias. According to Lindner et al.,* *^44^* *respondents were classified into two groups: early and late respondents. Late respondents were operationally and arbitrarily defined as the last half of respondents. Because there were 137 respondents in this study, 69 were identified as early respondents and 68 as late respondents. Hence, the independent samples t-test was then used to compare the two groups' responses to Likert scale questions.  The findings show that there is no significant difference in key metrics responses between early and late respondents.* *”*

**S11)** Please also explain how many responses are considered: "376 people", "137 questionnaires", or "100 questionnaires"? If either 137 or 100 questionnaires are used, the results may be inaccurate if only earlier replies are considered for examination. This would hinder possible differences among respondents and may potentially cause biases.

 I also did not understand the procedure followed while gathering questionnaires. The following two descriptions appear inconsistent:

*“As a result, the data collected reached 100 respondents in just a matter of days. Due to the limitations of the software features used, the distribution of the questionnaire was halted. As a result, all collected data were subjected to validity and reliability tests."*

*“The questionnaires were distributed electronically to the West Java MSME community. The questionnaires were returned by 376 people. The returned questionnaires were then sorted using predetermined criteria, yielding 137 questionnaires that met the criteria. However, due to the limitations of the software features used, this study only processed 100 questionnaires at random."*

 Response:

 We revised the explanation as follows:

*“* *The questionnaires were distributed to the members of the West Java MSME association's WhatsApp groups.* *The questionnaires were returned by 376 respondents. The returned questionnaires were then sorted using predetermined criteria, yielding 137 respondents that met the criteria* *.* *”*

*“* *Finally, we decided to distribute the electronic questionnaire Google Forms in May 2020 to the West Java MSME association's WhatsApp groups. In just a few days, the data collected reached 137 respondents who met the predetermined criteria, so all collected data were subjected to validity and reliability tests.”*

 As pointed out in the major comment, I do not agree with the use of 100 samples.

**S12)** Please add the corresponding population statistics to Table 7 and briefly discuss the possible biases, if any.

*“Table 7 displays the percentage of respondents for each indicator. As shown in Table 7, 98% of respondents have Islam as their religion (Muslim), 71% are female, and 88% are 26 years old or older."*

 Response:

 We tested the non-biased response, and the results are as follows:

*“* *We examined the responses of early and late respondents to see if there was any possibility of non-response bias. According to Lindner et al.,* *^44^* *respondents were classified into two groups: early and late respondents. Late respondents were operationally and arbitrarily defined as the last half of respondents. Because there were 137 respondents in this study, 69 were identified as early respondents and 68 as late respondents. Hence, the independent samples t-test was then used to compare the two groups' responses to Likert scale questions.  The findings show that there is no significant difference in key metrics responses between early and late respondents.* *”*

**S13)** Why do the authors not remove Christians from the selected 100 respondents? (Table 7 shows one Catholic and one Protestant.)

 Response:

 We state that

*“* *Non-Muslim respondents are involved because producing halal food regardless of the religion of the food producers* *.”*

**S14)** Please consider aligning digits in Table 8, e.g., "3.2225" maybe "3.223", etc.

 Response:

 We have revised the numbers

**S15)** Please specify the percentage selected by the authors as statistically significant (e.g., 5%) in Table 9.

 Response:

 We add the significance level of 5% in the body text and Table 12.

*“* *By using* *a significance level of 5%,* *the path coefficient will be significant if the t-value is larger than 1.65.* *^48^* *”*

**S16)** Please use a period instead of a comma as a decimal point. For example, the authors used both in the following paragraph (82,6% and 82.6%, respectively).

*“Besides, the R2 value of 0.826 for HA means that 82,6% of the variance in HA is influenced by KH and PB, with values β = 0,292 and 0,683 respectively. Hence, KH and PB have a strong and significant impact on the awareness of MSE entrepreneurs about the importance of halal products. According to Hair, et al.41 R2 values of 0.75, 0.50, or 0.25 for the endogenous construct, respectively, can be described as substantial, moderate, or weak. The R2 value of an endogenous latent variable (i.e., halal awareness) described by the two predictive constructs in this study is 82.6% (see Figure 2), which is substantial."*

*Response:*

 We use a period as a decimal point in the following paragraph

*“* *Besides, the R ^2^ value of 0.841 for HA means that 84.1% of the variance in HA is influenced by KH and PB, with values β = 0.169 and 0.791 respectively. Hence, KH and PB have a strong and significant impact on the halal awareness of MSE food producers.”*

*“* *According to Hair, et al.,* *^41^* *R ^2^ values of 0.75, 0.50, or 0.25 for the endogenous construct, respectively, can be described as substantial, moderate, or weak. The R ^2^ value of an endogenous latent variable (i.e., halal awareness) described by the two predictive constructs in this study is 84.1% (see* *Figure 3* *), which is substantial.* *”*

**S17)** The intended meaning of the following sentence is ambiguous.

*“The finding that knowledge of halal has a significant impact on knowledge of halal is different from the finding of Giyanti and Indriastiningsih ^14^"*

 The phrase “ *knowledge of halal has a significant impact on knowledge of halal*” is not clear. Please consider rewording it.

 Please also add a period at the end of this sentence.

 Response:

 We revised the explanation as follows:

*“The finding that knowledge of halal has a significant impact on halal awareness is different from the finding of Giyanti and Indriastiningsih.* *^16^* *”*

**S18)** I do not understand the following paragraph. Did the MSE find it beneficial or unnecessary to obtain halal certificates?

*“The findings also show that MSEs are aware of the benefits of the halal certificate. Based on our observation, we find that they do not require a halal certificate because they have satisfied with the sales/performance that has been achieved."*

 Response:

 The explanation has been revised as follows:

*“* *The findings also indicate that MSE food producers are aware of the benefits of the halal certificate. However, our observation revealed that they perceive obtaining a halal certificate as prohibitively expensive. It will cause the halal-certified product's selling price to rise. They are concerned that increasing selling prices will reduce sales. As a result, they perceive that obtaining a halal certificate is unnecessary. In any case, their business is running smoothly without the halal certificate. It aligns with the findings of Prabowo et al.* *^13^* *”*

 Similarly, I also do not understand why the authors conclude “ *[h]alal certificates will therefore increase sales and revenue*” in the following context? Please consider a more logical construction of the sentence.

*“They are unaware that halal certificates will increase consumer trust and help food businesses compete more effectively. Halal certificates will therefore increase sales and revenue."*

 Response:

 We delete this statement due to the contradiction with the previous statement.

**S19)** Please explain the relationship between respondents of this study and “ *street vendors around Universitas Islam Bandung (Unisba)*” that is mentioned in the “Discussion” section.

*“Socialization and training on halal awareness, halal guarantee system, and halal certification for street vendors around Universitas Islam Bandung (Unisba) which is located at Bandung, the Capital of West Java Province Indonesia have been conducted by Oemar et al.13 The trainees gain a better understanding and awareness of halal food as a result of the training. Consequently, all trainees intend to obtain a Halal Certificate."*

 Response:

 The explanation has been revised as follows.

*“* *Oemar et al.* *^17^* *proved that socialization and training on halal awareness, halal assurance systems, and halal certification increase the understanding and awareness about halal-certified food so that all participants intend to obtain a Halal Certificate after completing the training.”*

**S 20)** Please recheck the references carefully. For example, you can quote my work (reference 9), but the publication year, pages, etc., are incorrect.

 Response:

 The reference writing has been corrected.
